# The Complete Sequence of the *Acacia ligulata* Chloroplast Genome Reveals a Highly Divergent *clpP1* Gene

**DOI:** 10.1371/journal.pone.0125768

**Published:** 2015-05-08

**Authors:** Anna V. Williams, Laura M. Boykin, Katharine A. Howell, Paul G. Nevill, Ian Small

**Affiliations:** 1 Australian Research Council Centre of Excellence in Plant Energy Biology, The University of Western Australia, Crawley, Western Australia, Australia; 2 Botanic Gardens and Parks Authority, Kings Park and Botanic Garden, Fraser Avenue, Kings Park, Western Australia, Australia; 3 School of Plant Biology, The University of Western Australia, Crawley, Western Australia, Australia; 4 Centre of Excellence in Computational Systems Biology, The University of Western Australia, Crawley, Western Australia, Australia; Academia Sinica, TAIWAN

## Abstract

Legumes are a highly diverse angiosperm family that include many agriculturally important species. To date, 21 complete chloroplast genomes have been sequenced from legume crops confined to the Papilionoideae subfamily. Here we report the first chloroplast genome from the Mimosoideae, *Acacia ligulata*, and compare it to the previously sequenced legume genomes. The *A*. *ligulata* chloroplast genome is 158,724 bp in size, comprising inverted repeats of 25,925 bp and single-copy regions of 88,576 bp and 18,298 bp. *Acacia ligulata* lacks the inversion present in many of the Papilionoideae, but is not otherwise significantly different in terms of gene and repeat content. The key feature is its highly divergent *clpP1* gene, normally considered essential in chloroplast genomes. In *A*. *ligulata*, although transcribed and spliced, it probably encodes a catalytically inactive protein. This study provides a significant resource for further genetic research into *Acacia* and the Mimosoideae. The divergent *clpP1* gene suggests that *Acacia* will provide an interesting source of information on the evolution and functional diversity of the chloroplast Clp protease complex.

## Introduction

The Leguminosae (Fabaceae) are a large and economically important family of flowering plants. The family is separated into a number of subfamilies, with Papilionoideae and Mimosoideae being the most species-rich. The Papilionoideae has been the best studied of these subfamilies due to the fact that it includes a large number of agriculturally important species, such as soybean (*Glycine max* L.), chickpea (*Cicer arietinum* L.), the common bean (*Phaseolus vulgaris* L.) and mungbean (*Vigna radiata* L.).

The Mimosoideae includes genera such as *Mimosa*, *Inga* and *Acacia*. The genus *Acacia* (*sensu stricto*) is found across tropical, subtropical, warm temperate and arid climates. It occurs predominantly in Australia, although several species also occur in Southeast Asia and Madagascar [1]. With over 1,000 species, *Acacia* is the largest angiosperm genus in Australia [2]. *Acacia* species play an important ecological role both as a dominant component of many vegetation classes in Australia [3], particularly in the arid/semi-arid interior, and also internationally as invasive species [4–6]. Many Austalian acacias are also important sources of wood and wood products and are widely grown in the tropics and sub-tropics [7]. Previous genetic research on *Acacia* has focused largely on informing conservation and agro-forestry management, for example by identifying provenances for seed sourcing [8], examining mating systems and the level and distribution of genetic variation within species [9–11], establishing phylogeographic patterns [12], enhancing species identification through DNA barcoding [6, 13], and clarifying species relationships in phylogenetic studies [14–19].

In recent years, the benefits of whole genome sequencing to conservation and restoration genetics have become increasingly clear. These benefits include large-scale development of both neutral and adaptive markers and larger datasets for increased phylogenetic resolution [20–23]. Prior to the development of next-generation sequencing technologies, the time and cost associated with sequencing an entire genome was impractical for non-model species. However, in the last decade, the development of high throughput technologies has made whole genome sequencing increasingly practical and cost-effective, notably via high-throughput shallow sequencing of total DNA [24, 25].

To date, approximately 530 complete chloroplast genomes have been sequenced (see http://www.ncbi.nlm.nih.gov/genomes/GenomesGroup.cgi?taxid=2759&opt=plastid), with 21 of these belonging to the Leguminosae (all 21 are Papilionoideae). The typical chloroplast genome comprises two inverted repeats (IRs) separated by a small single copy (SSC) and a large single copy (LSC) region [26]. In general, chloroplast genomes range in size from 120-160-kb and include 120–130 genes, many of which are essential for photosynthesis. The chloroplast’s role in photosynthesis has resulted in these features being highly conserved [27–29].

Compared to this typical chloroplast genome, many Papilionoideae chloroplast genomes display significant rearrangements, including the inversion of a 50-kb region of the LSC [30, 31], and the loss of one inverted repeat copy [32]. These features, as well as transfer of the *rpl22* gene to the nucleus [33, 34], and intron loss in the *clpP* and *rps12* genes [35, 36], have been well studied and their presence/absence mapped onto the current Leguminosae phylogeny [36].

Unlike Papilionoideae, Mimosoideae appears to display neither loss of the inverted repeat [37], nor the 50-kb inversion [30], however, no complete Mimosoideae chloroplast genomes have yet been sequenced. Here we report the complete chloroplast genome sequence of *Acacia ligulata*, a widespread species found throughout arid and semi-arid Australia. We discuss the chloroplast genome structure of *A*. *ligulata* including its gene content, inverted repeat organisation and repeat structure, and compare these with other legume chloroplast genomes. We also investigated the functionality of the *A*. *ligulata clpP1* gene by exploring the transcript’s ability to be spliced, and the conservation of the catalytic triad.

## Results and Discussion

### Sequencing and assembly

**Fig 1:**
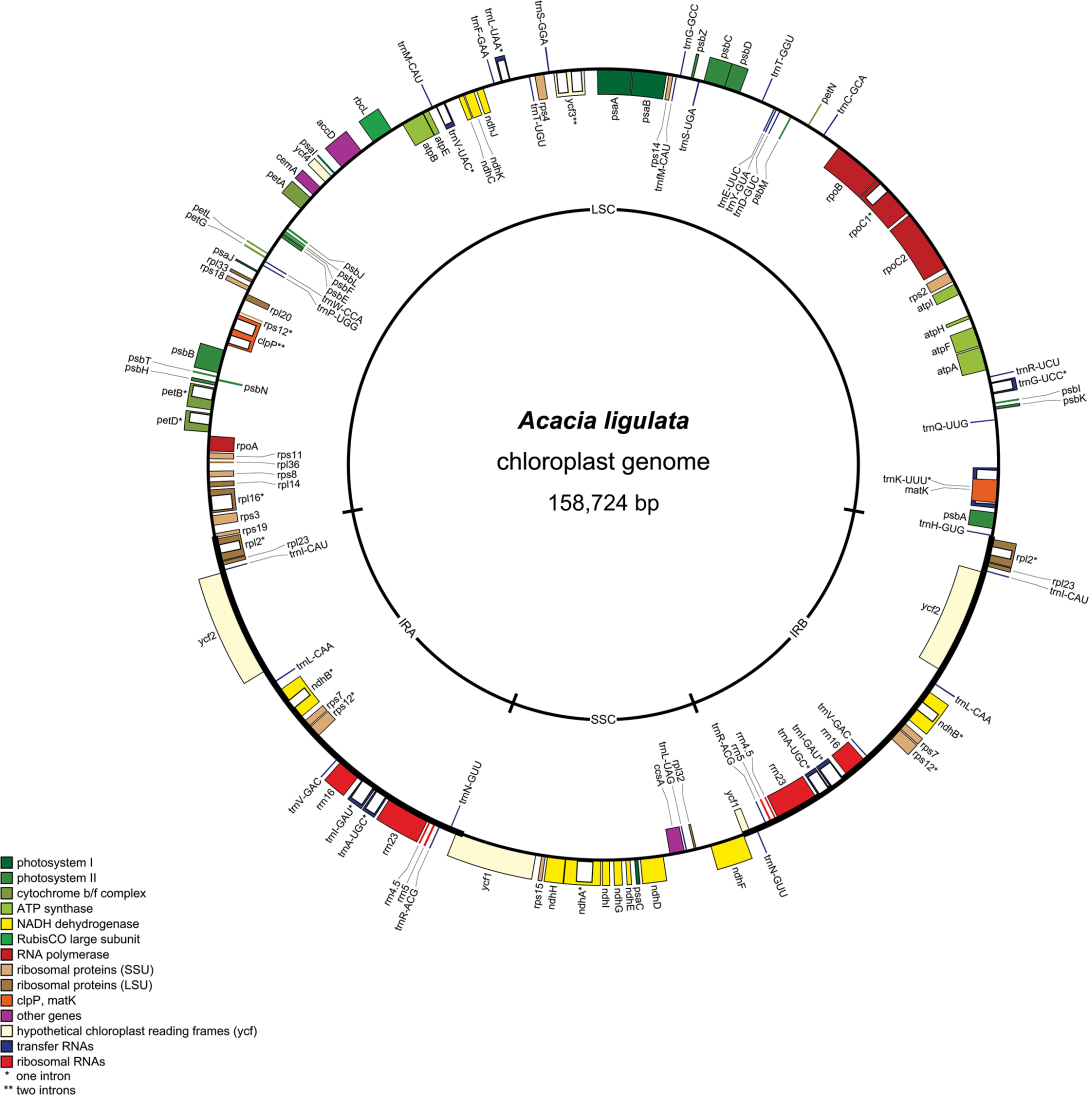
Genome Map of the *Acacia ligulata* Chloroplast. Genes shown on the inside of the circle are transcribed in the clockwise direction and those shown on the outside of the circle are transcribed in the anticlockwise direction. Genes marked with an asterisk contain introns, with the introns indicated by clear boxes. The legend indicates the functional group to which each gene belongs. The figure was generated with OrganelleGenomeDRAW [79].

Dried herbarium material of a specimen of *Acacia ligulata* Benth. was used for DNA extraction. Illumina sequencing of a library prepared from total DNA produced 2,216,882 paired-end reads with a read length of 100 nt. 5.26% of reads were assembled into 23 contigs showing homology to legume plastid DNA. Gaps between contigs were then filled by PCR amplification and Sanger sequencing. The complete assembled chloroplast genome of *A*. *ligulata* is typical in its general structure with a pair of IRs of 25,925 bp, an LSC of 88,576 bp and an SSC of 18,298 bp (Fig 1). Thus, unlike the chloroplast genomes of many of the Papilionoideae, the *A*. *ligulata* genome has inverted repeats and no inversions within the LSC. The total size of the *A*. *ligulata* chloroplast genome is 158,724 bp, 49.4% of which is non-coding DNA. The GC content for the whole genome is 36.2%, while that of the protein-coding, rRNA and tRNA genes is 37.4%, 55.3% and 53.2%, respectively. These values are similar to those in other Leguminosae genomes (see Table 1 for those used in our comparisons).

**Table 1:** GenBank Accession Numbers and References for All Taxa Used in the Phylogenetic and Genomic Comparison of *Acacia ligulata*.

Species	Family	GenBank accession	Genome size (bp)	Reference
*Cicer arietinum*	Leguminosae	NC_011163	125,319	[36]
*Glycine canescens*	Leguminosae	KC893635	152,518	Unpub.
*Glycine cyrtoloba*	Leguminosae	KC893632	152,381	Unpub.
*Glycine dolichocarpa*	Leguminosae	KC893636	152,804	Unpub.
*Glycine falcata*	Leguminosae	KC563637	153,023	Unpub.
*Glycine max*	Leguminosae	NC_007942	152,218	[38]
*Glycine soja*	Leguminosae	KF611800	152,217	Unpub.
*Glycine stenophita*	Leguminosae	KC893634	152,618	Unpub.
*Glycine syndetika*	Leguminosae	KC893638	152,783	Unpub.
*Glycine tomentella*	Leguminosae	KC893633	152,728	Unpub.
*Lathyrus sativus*	Leguminosae	NC_014063	121,020	[34]
*Lotus japonicus*	Leguminosae	AP002983	150,519	[39]
*Lupinus luteus*	Leguminosae	NC_014063	151,894	[40]
*Medicago truncatula*	Leguminosae	NC_003119	124,033	Unpub.
*Millettia pinnata*	Leguminosae	NC_016708	152,968	[41]
*Phaseolus vulgaris*	Leguminosae	NC_009259	150,285	[42]
*Pisum sativum*	Leguminosae	NC_014057	122,169	[34]
*Trifolium subterraneum*	Leguminosae	EU849487	144,763	[43]
*Vigna angularis*	Leguminosae	AP012598	151,683	Unpub.
*Vigna radiata*	Leguminosae	NC_013843	151,271	[44]
*Vigna unguiculata*	Leguminosae	JQ755301	152,415	Unpub.
*Pyrus pyrifolia*	Maleae	NC_015996	159,922	[45]
*Morus indica*	Moraceae	DQ226511	158,484	[46]
*Castanea mollissima*	Fagaceae	NC_014674	160,799	[47]
*Cucumis sativus*	Cucurbitaceae	DQ119058	155,527	[48]
*Eucalyptus globulus*	Myrtaceae	KC180787	160,267	[49]

### Genome content and order

**Fig 2:**
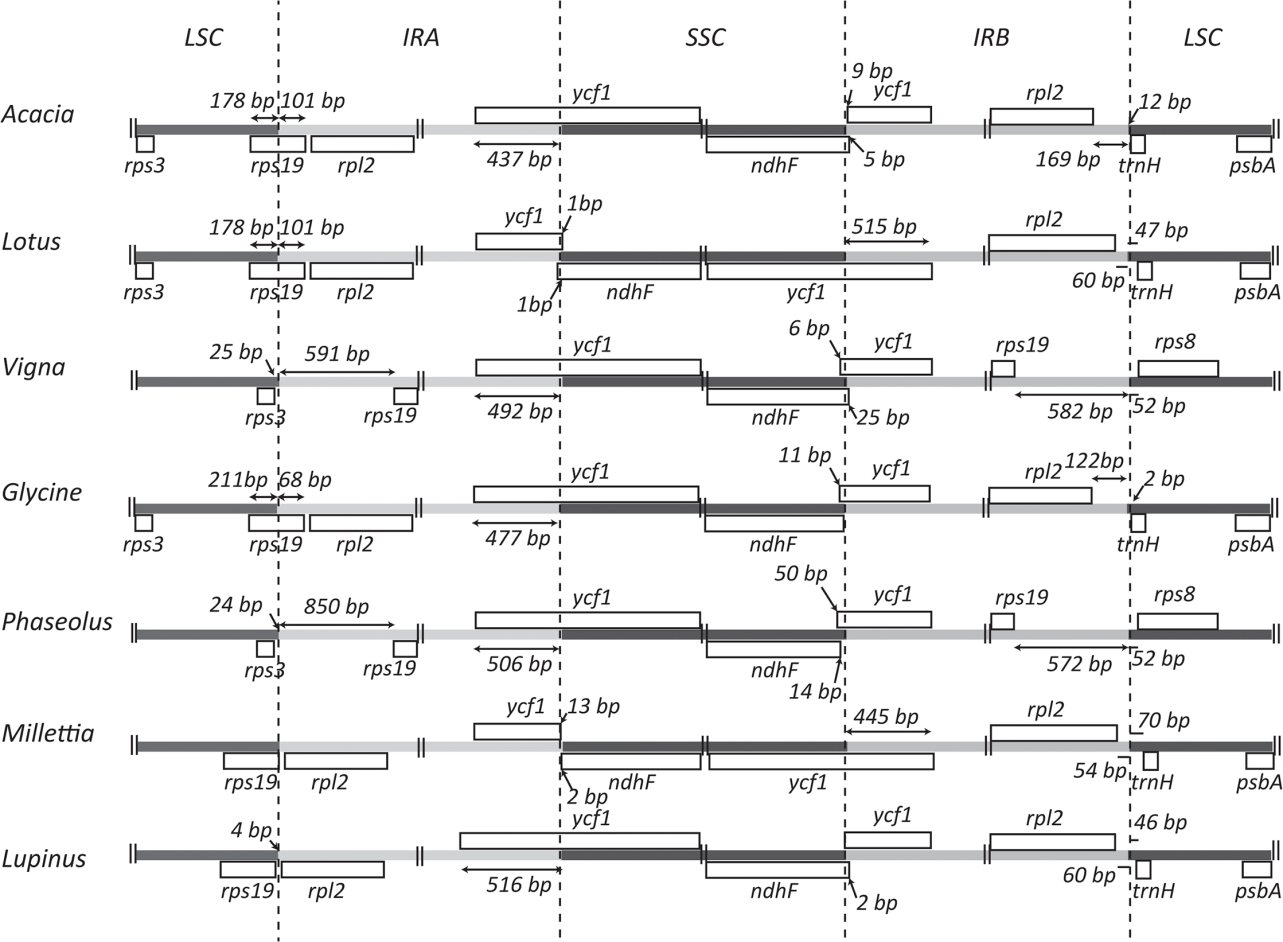
Structure of the LSC/IR junction regions in legume genera. Protein coding regions are indicated by grey boxes with genes below the line being transcribed right to left and those below the line transcribed left to right. The number of base pairs between the end of the gene and the IR is indicated for genes on either side of the junction, unless the junction coincides with the end of a gene.

The *A*. *ligulata* chloroplast genome contains 109 unique genes, including 76 unique protein-coding genes, 4 unique rRNA genes and 29 unique tRNA genes. As is seen throughout the Leguminosae, the *rpl22* gene is absent from the *A*. *ligulata* plastid genome following an ancient transfer to the nuclear genome [33]. The inverted repeat of the *A*. *ligulata* chloroplast genome results in the complete duplication of the *rpl2*, *rpl23*, *ycf2*, *ndhB* and *rps7* genes, as well as exons 1 and 2 of *rps12*, all four rRNA genes and seven tRNA genes. As is also seen in those Leguminosae species that retain their inverted repeat, the IR of the *A*. *ligulata* chloroplast runs roughly 450 bp into the *ycf1* gene. This feature has been shown to distinguish legume chloroplasts from many other angiosperms, which typically have 1,000 bp or more of the *ycf1* gene included in their IR [38]. Of those legumes that do retain the inverted repeat, that of *A*. *ligulata* is larger than in *Lupinus*, *Glycine*, *Lotus* and *Millettia*, but smaller than in *Phaseolus* and *Vigna* (Fig 2). The *rps19* gene of *A*. *ligulata* is found partially within the IR, with 101 bp being repeated. This is consistent with *Glycine* and *Lotus* that also display partial duplication of the *rps19* gene. However, this feature varies throughout the Leguminosae, with the duplication of the entire gene in *Phaseolus* and *Vigna*, while *rps19* is not within the IR for *Millettia* and *Lupinus*.

Eleven protein-coding genes and seven tRNA genes contained at least one intron, with *clpP1*, *rps12* and *ycf3* each containing two introns. This is in contrast to *Cicer arietinum*, *Medicago truncatula*, *Trifolium subterraneum*, *Pisum sativum* and *Lathyrus sativus*, all of which have lost an intron in both *clpP1* and *rps12* [36]. The largest intron was found in *trnK-UUU* (2,544 bp), spanning the entire *matK* gene, whilst *trnL-UAA* contains the smallest intron (543 bp). Two sets of open reading frames overlap: *atpA* and *atpE* overlap by four nucleotides whilst *psbC* and *psbD* overlap by 17 nucleotides, taking the start codon of *psbC* to be the GTG codon at position 36,432, based on the results on *psbC* translation in tobacco [50].

### Repeat content

**Fig 3:**
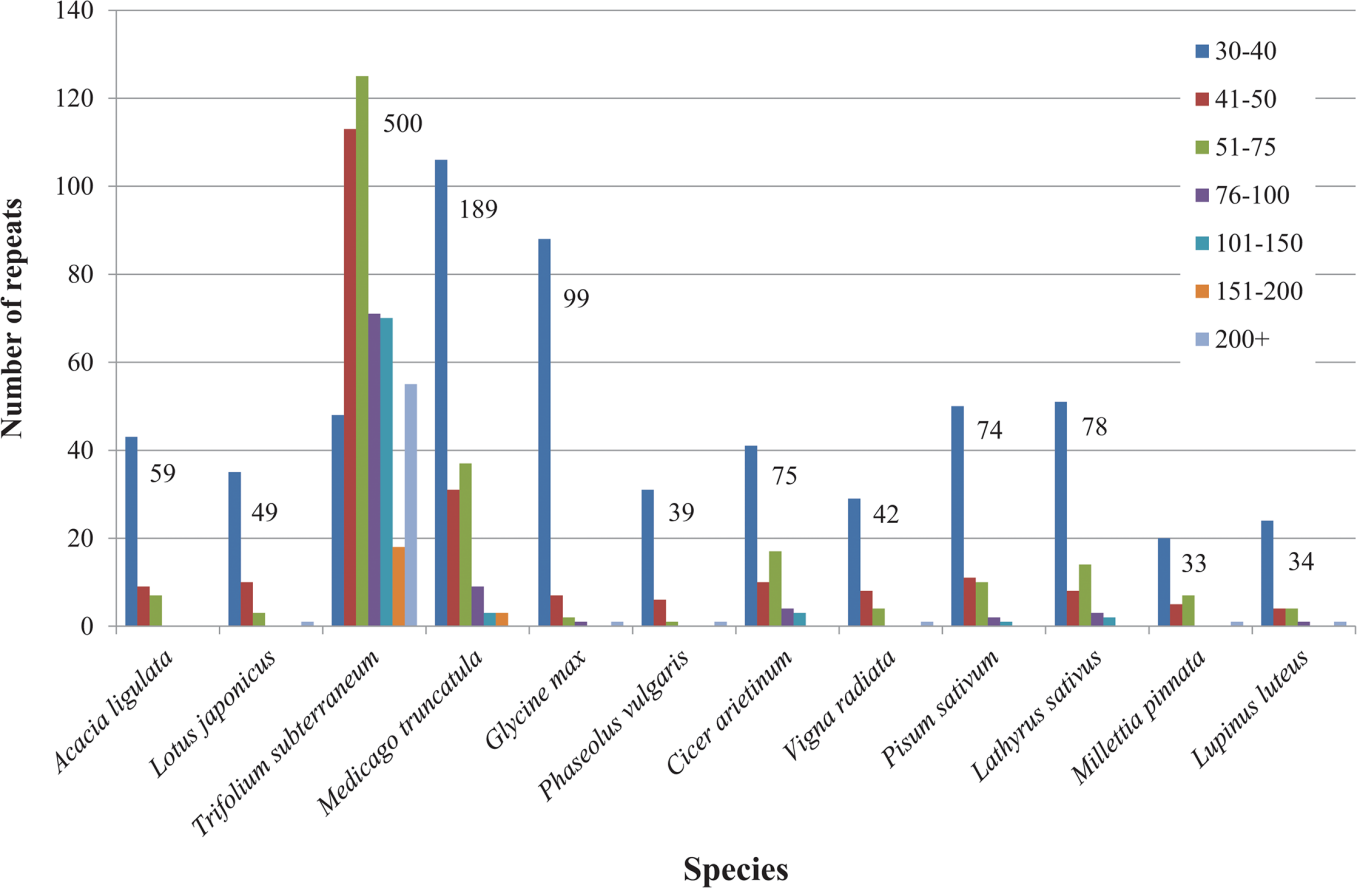
*Acacia ligulata* Chloroplast Genome Repeat Content Compared to that of Other Legume Genomes. Repeats are separated into groups according to their size, and the total number of repeats is shown above the bars.

The 59 sets of direct and indirect repeats of 30 bp or longer in the *A*. *ligulata* chloroplast genome are listed in S1 Table (not including the large IRs). These include 29 forward repeats, seven reverse repeats, four complementary repeats and 19 palindromic repeats. Repeats were found in the *rpl16*, *ndhA*, *ycf3* and *clpP1* introns, and in the *accD*, *psaA* and *psaB* genes. Compared to other legumes, *A*. *ligulata* has a typical repeat content. The *Trifolium subterraneum* plastid genome contains by far the greatest number of repeats with 500 repeats in total, while *Millettia pinnata* and *Lupinus luteus* have the fewest, with 33 and 34 repeats, respectively (Fig 3).

One of these repeats, in the *psbJ-petA* spacer, is a tandem duplication of 60 bp. This is shorter than the longest tandem repeats found in other legumes: for example, some tandem repeats in *Cicer arietinum*, *Medicago truncatula* and *Trifolium subterraneum* are well over 100 bp in length. The *A*. *ligulata* chloroplast genome contains another 31 tandem repeats of 10 bp or more in length (S2 Table). Ten were found within genes, including sets in the *ndhA*, *atpF* and *clpP1* introns. The remaining repeats were found within intergenic spacer regions. Two sets of tandem repeats observed in *A*. *ligulata* are also found in other legumes: repeat 13 is also in the *rps12-trnV* spacer regions of *Lotus japonicus*, *Millettia pinnata* and *Lupinus luteus*, whereas repeat 21 is also in the *ycf2* genes of *Millettia pinnata* and *Lupinus luteus*.

### Phylogenetic analysis

**Fig 4:**
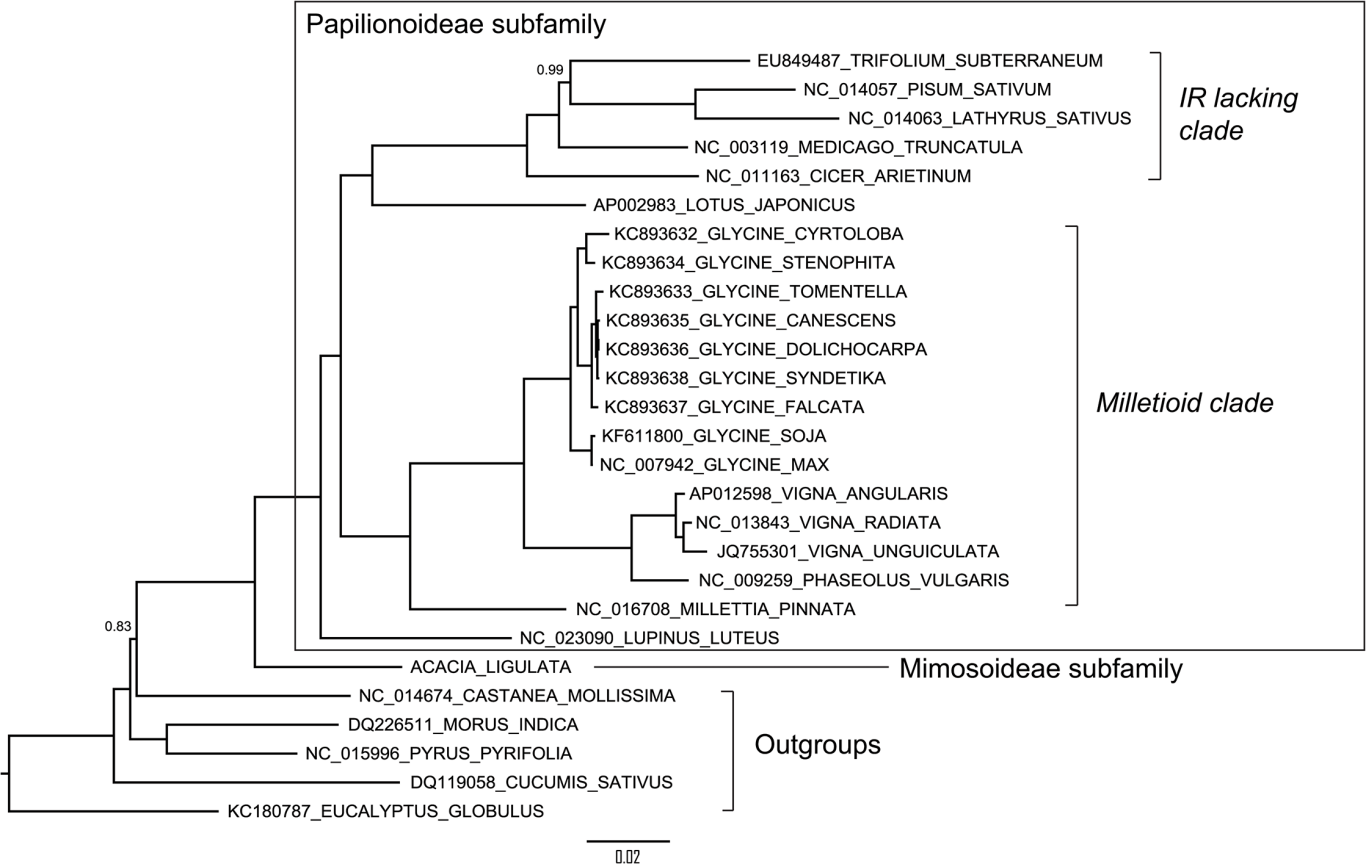
Phylogenetic Tree Constructed from 74 Concatenated Chloroplast Genes Showing the Position of *Acacia ligulata*. Phylogenetic reconstruction was performed using MrBayes with a General Time Reversible model with gamma and invariant sites. Posterior probabilities are indicated above the branches where they differ from 1.

Phylogenetic reconstruction of the 74 concatenated *A*. *ligulata* chloroplast genes, with introns removed, supported previous phylogenetic hypotheses based on both genome rearrangement [36] and the *matK* gene [32, 51], that place *Acacia* sister to a clade containing all the Papilionoideae legume taxa. *Lupinus* is sister to a clade containing two subclades, one containing *Cicer*, *Medicago*, *Trifolium*, *Pisum* and *Lathyrus*, and a second containing *Millettia*, *Phaseolus*, *Vigna* and *Glycine* (Fig 4). All nodes are strongly supported and the phylogeny generated from concatenated genes of the chloroplast genomes is congruent with all but 7 of the 74 trees built from individual chloroplast genes (data not shown).

Although not included in the concatenated phylogeny due to its loss in *Pisum sativum*, a phylogeny was also built for the *ycf4* gene (S1 Fig). The *ycf4* gene has previously been identified as a region of hypermutation in the Papilionoideae. Although this gene is typically 555 bp long, it has gained an additional several hundred bp in *Glycine max*, *Lotus japonicus* and *Lathyrus* [34, 39, 52]. *Acacia ligulata* does not display an elevated rate of divergence in this gene, as shown by the short branch length similar to those in the outgroup *ycf4* genes.

### Divergence in *clpP1*

**Fig 5:**
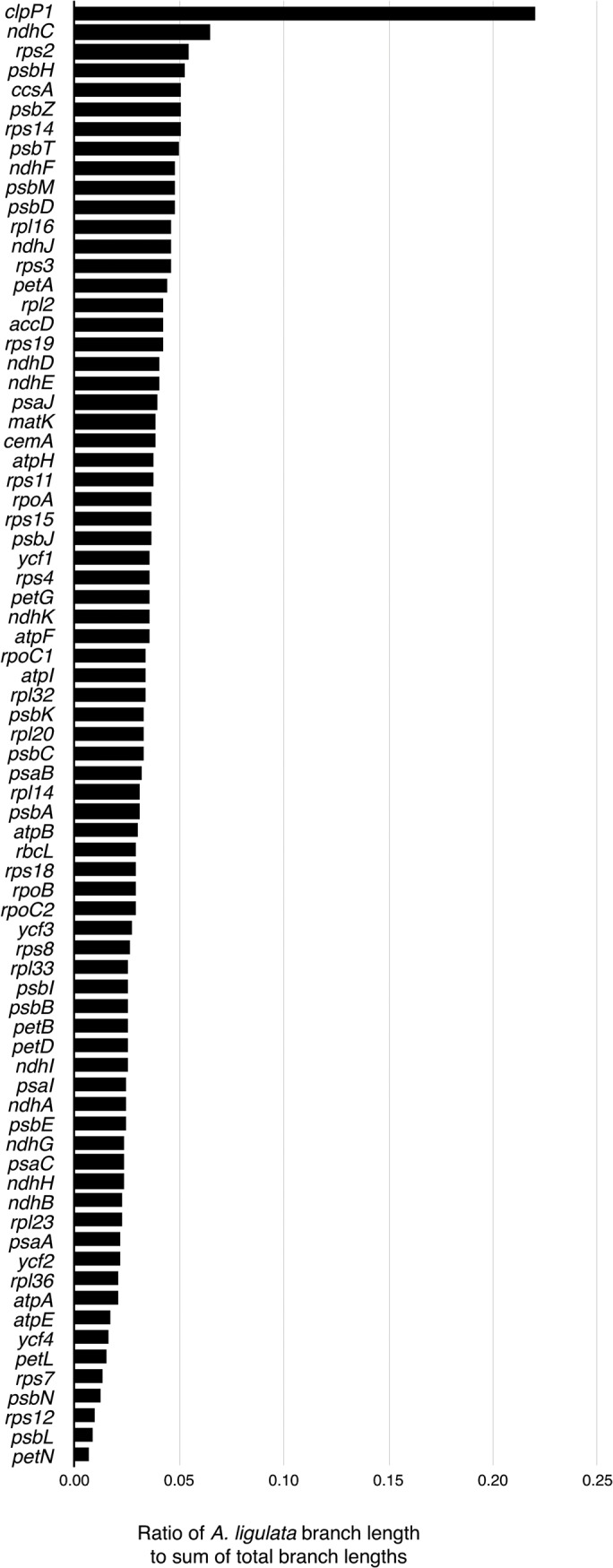
Relative branch lengths leading to *Acacia ligulata* in different gene trees. Phylogenetic reconstructions were performed separately for each individual gene alignment using MrBayes with a General Time Reversible model with gamma and invariant sites. The bar chart indicates the proportion of the total branch length in each tree contributed by the branch leading to *Acacia ligulata*.

**Fig 6:**
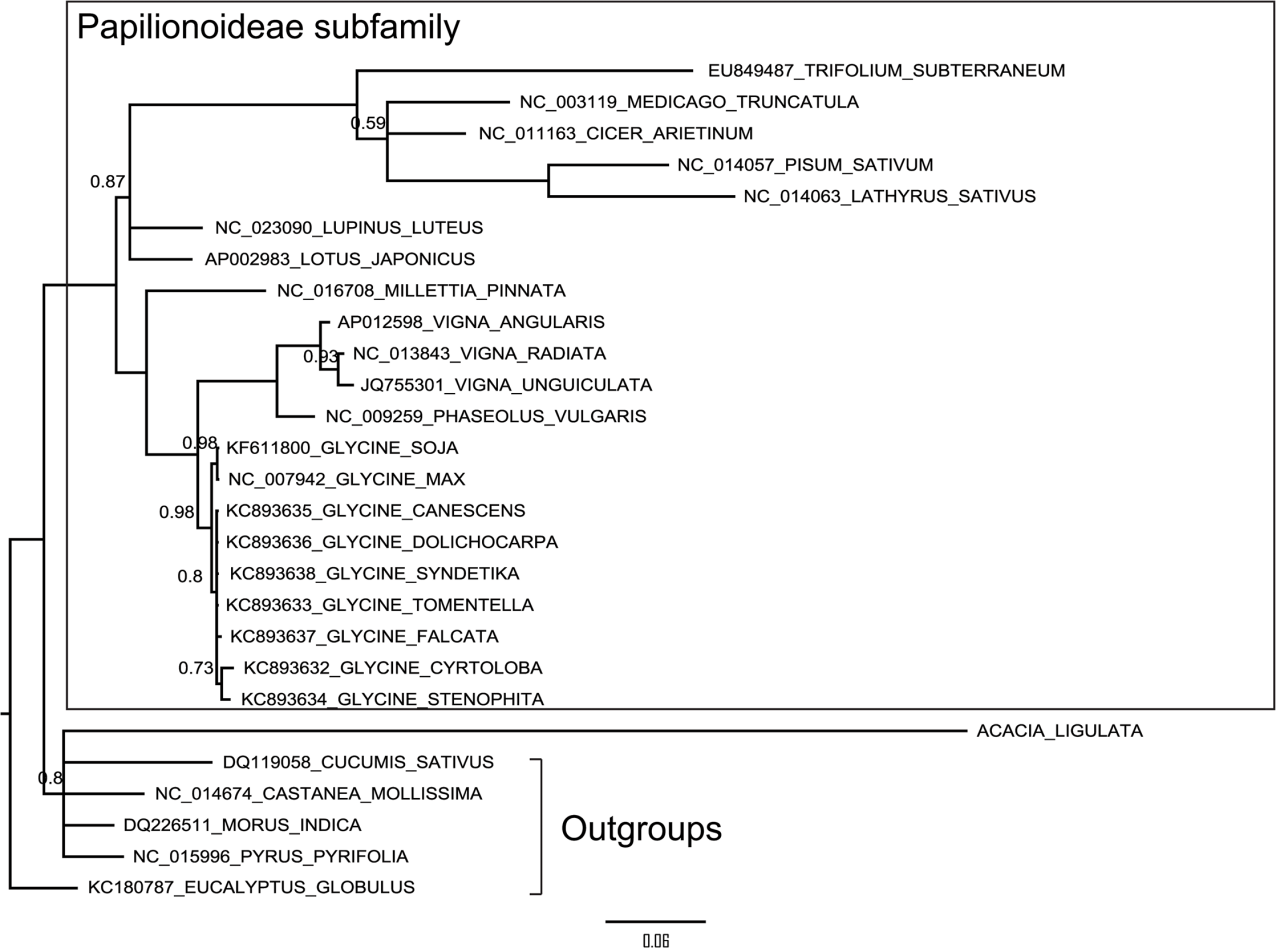
Phylogenetic Tree of the *clpP1* Gene Showing High Divergence in *Acacia ligulata*. Phylogenetic reconstruction was performed using MrBayes with a General Time Reversible model with gamma and invariant sites. Posterior probabilities are indicated above the branches where they differ from 1.

In contrast to *ycf4*, the *clpP1* gene is highly divergent in *Acacia*, as indicated by the unusually long branch length leading to *A*. *ligulata* in the tree based on *clpP1* sequences (Fig 5). In order to determine the selective pressures influencing the divergence of the *A*. *ligulata clpP1* gene, the non-synonymous versus synonymous nucleotide substitution ratio (dN/dS) was calculated using an alignment of *clpP1* coding sequences (S2 Fig). A model using one dN/dS ratio across the *clpP1* phylogeny (Fig 6) was compared to a model in which a separate ratio was calculated for the *A*. *ligulata* branch. The two-ratios model was found to be a significantly better fit to our nucleotide data than the one-ratio model (likelihood ratio test, *P <* 0.00001). In this model, the branches leading to the *clpP1* genes of all species excluding *A*. *ligulata* were found to exhibit a low dN/dS ratio (0.30), indicative of purifying selection, as would be expected for such a highly conserved gene. In contrast, the branch leading to *A*. *ligulata* showed a dN/dS ratio (1.07) statistically indistinguishable from that in a model where the dN/dS ratio was fixed as 1 (likelihood ratio test, *P* > 0.99). This suggests that the *clpP1* sequence in *A*. *ligulata* may not be under selection at all. An absence of detectable selection is generally considered a strong sign of a pseudogene [53]; however, none of the sequence changes lead to frameshifts or premature stop codons that would clearly indicate that *clpP1* is a pseudogene.

**Fig 7:**
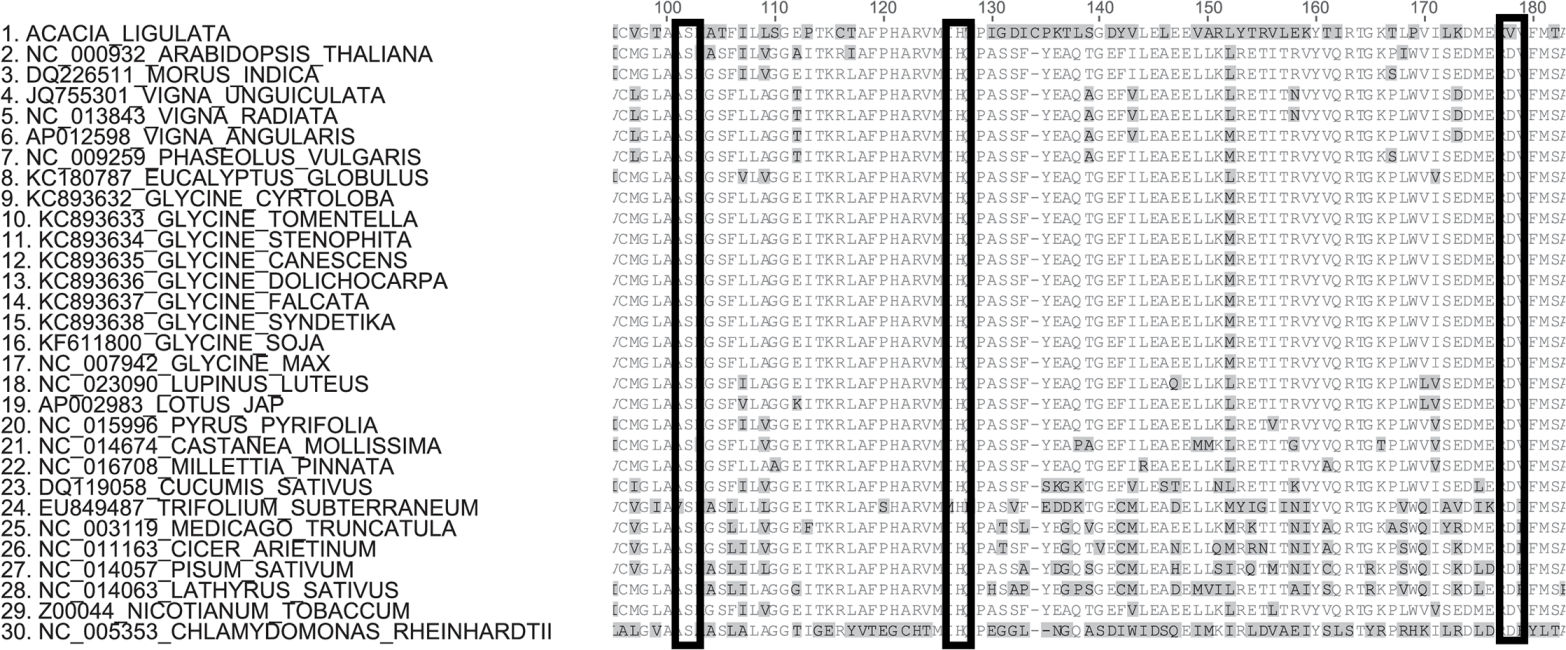
Alignment of a Region of the ClpP1 Protein Sequence. Alignment of a portion of the ClpP1 protein sequence from *Arabidopsis thaliana*, *Acacia ligulata*, other legumes and outgroups. The three residues of the catalytic triad at amino acid positions 102, 127 and 178 are indicated by black boxes. They are invariant apart from the mutation of aspartate 178 to valine in *A*. *ligulata*.

**Fig 8:**
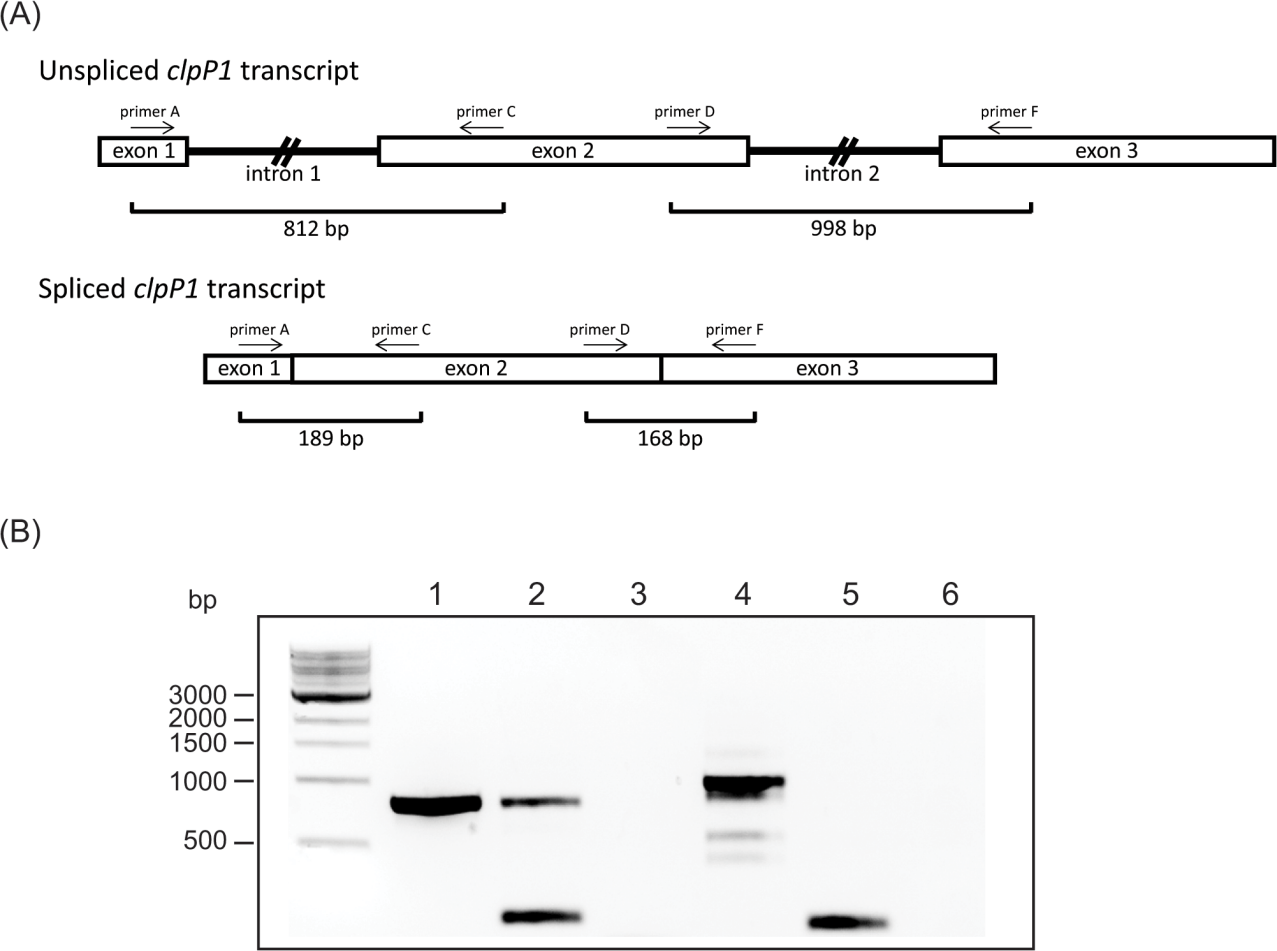
Splicing of the *Acacia ligulata clpP1* Transcript. (A) Schematic representation of the *clpP1* transcript showing unspliced and spliced forms. Primer positions are indicated by arrows and the predicted size of PCR products are shown. (B) Ethidium bromide stained 1.0% agarose gel showing PCR amplified products of (1) *Acacia ligulata* DNA with Primer A + Primer C; (2) *Acacia ligulata* cDNA with Primer A + Primer C; (3) negative control for Primer A + Primer C; (4) *Acacia ligulata* DNA with Primer D + Primer F; (5) *Acacia ligulata* cDNA with Primer D + Primer F; and (6) negative control for Primer D + Primer F.

The *clpP1* gene encodes a serine protease that is a subunit of the Clp protease [54]. Deletion of the *clpP1* gene in both tobacco and *Chlamydomonas reinhardtii* shows that the gene product is absolutely essential [55–57] and indeed it is one of the few genes consistently conserved in non-photosynthetic parasitic or mycoheterotrophic plants that have greatly reduced chloroplast genomes [58–61]. The poor conservation of this gene in *A*. *ligulata* was therefore a surprise. Sequence alignments revealed that a hitherto invariant aspartate (part of the typical protease catalytic triad) has been mutated to a valine in *A*. *ligulata* (Fig 7). This mutation cannot be reversed by RNA editing and would imply that the gene product cannot be catalytically active. Mutation of the corresponding aspartate to alanine in bacterial ClpP1 orthologues eliminates proteolytic activity [62]. To verify that the *clpP1* gene is actually expressed, we analysed *A*. *ligulata clpP1* transcripts by RT-PCR (Fig 8). Transcripts were easily detected and both introns can be correctly spliced out (verified by sequencing of the products obtained using cDNA as a template), although many transcripts retain intron 1 (Fig 8B). Despite the divergent sequence, this suggests that the *clpP1* protein might still be synthesised. In plastids, the Clp complex consists of a heterotetradecameric core composed of two rings of seven subunits [63]. The R-ring consists of three copies of catalytically active ClpP1 (the only subunit encoded by the plastid genome) and single copies of the catalytically inactive ClpR3, ClpR4, ClpR5 and ClpR6 subunits [64]. The P-ring consists of ClpP3, ClpP4, ClpP5, ClpP6 in the ratio 1:2:3:1 [64]. It is possible therefore, that the *A*. *ligulata* plastid *clpP1* gene product assembles into a Clp complex whose proteolytic function is assured by nucleus-encoded ClpP subunits in the P-ring. However, loss of the ClpP1 active site would completely remove the catalytic activity from the R-ring. To our knowledge, the effects of a loss of activity of a specific ClpP subunit (as opposed to loss of the whole subunit) has not been tested in plants. Lack of expression of individual ClpP subunits leads to severe phenotypes (lethal in the case of ClpP1, ClpP4 and ClpP5; plants lacking ClpP3 can grow heterotrophically, but very slowly; reviewed in [63]).

### Search for a nuclear *clpP1* gene

It seemed possible that the *clpP1* gene has been transferred to the nucleus, and is functionally expressed from this new location in *A*. *ligulata*, as suggested in other rare cases where the chloroplast gene appears to be non-functional [65]. In order to identify any potentially nuclear *clpP1* sequences, raw reads were compared to the chloroplast *clpP1* gene of *Lupinus luteus*, the closest relative to *Acacia* with an available *clpP1* sequence. Given that a functional transfer of a chloroplast sequence to the nuclear genome would most likely require loss of the two introns, the spliced *Lupinus luteus* sequence was used for the search. No reads aligning across the splice junctions were found (Table 2). So that the likelihood of identifying nuclear *clpP1* reads given the low coverage expected could be ascertained, this analysis was repeated using nuclear *CLP* gene sequences from *Medicago truncatula* and *Glycine max* (the closest relatives of *Acacia* with sequenced nuclear genomes) as reference sequences. For some of the genes, reads potentially encoding Clp subunits were identified (Table 2). These reads confirm that the *A*. *ligulata* nuclear genome does encode subunits for a probable plastid Clp protease, but the low coverage precludes us from concluding whether or not these nuclear genes include a *clpP1* paralogue.

**Table 2:** Results of searches for nucleus-encoded subunits of a plastid Clp complex in the *Acacia ligulata* sequences.

Accession	Species	Gene	Length (in bp)	No. of hits	Min. identity	E-value range	% coverage
NC_023090	*L*. *luteus*	*clpP1*	591	0			
XM_003624370	*M*. *truncatula*	*CLPP3*	1165	1	80%	0.086	8.67%
XM_003612554	*M*. *truncatula*	*clpP4*	1172	3	81%	1.3 – 9e-05	17.23%
XM_003591344	*M*. *truncatula*	*CLPP5*	1185	0			
XM_003625930	*M*. *truncatula*	*CLPP6*	1163	3	89%	1e-06 – 4e-13	21.66%
XM_003592441	*M*. *truncatula*	*clpR1*	1564	1	91%	1e-07	6.65%
XM_003608743	*M*. *truncatula*	*CLPR2*	1026	0			
XM_003626156	*M*. *truncatula*	*CLPR3*	1416	0			
XM_006600793	*G*. *max*	*ClpR4*	1286	1	90%	1e-25	7.85%

The choice of and nomenclature of these subunits follows the current understanding of the structure of the chloroplast Clp complex [63].

## Conclusions

Investigations of the *A*. *ligulata* chloroplast genome revealed that it resembles a typical angiosperm chloroplast genome, with respect to structure and gene content. The large inversions and deletions observed in the Papilionoideae are not present in the *A*. *ligulata* chloroplast genome. Our well-resolved phylogenetic analysis supports existing proposed phylogenies for the Leguminosae. The most unusual feature of the genome is the highly divergent *clpP1* gene. Our analysis of this gene suggests that the gene is expressed, but the protein product may not be catalytically active.

## Methods

### DNA sequencing

Dried phyllode material was obtained from a specimen of *Acacia ligulata* Benth. (Fabaceae) held at the Western Australian Herbarium (voucher number: PERTH07807864; collected at Lorna Glen, Western Australia, in 2006). Total genomic DNA was extracted using a CTAB protocol [11]. DNA quantity and quality were assessed using a NanoDrop spectrophotometer (ND-1000; Thermo Fisher Scientific, USA), and agarose gel electrophoresis, respectively. Genome library preparation was performed using a Nextera DNA Sample Preparation Kit (Illumina, San Diego, USA), following the manufacturer’s directions. The library was prepared for sequencing using the cBOT cluster generation system and PE V3 flow cell and cluster chemistry (Illumina). The library was sequenced on a single lane in paired-end mode using the HiSeq2000 platform and V3 SBS kit (Illumina). Library preparation and sequencing were both performed at the Ramaciotti Centre for Gene Function Analysis (Sydney, Australia; http://devspace.ddtoo.com/).

### Genome assembly

Overlapping paired-end reads were merged using the software FLASH version 1.2.7 [33] and merged reads were assembled using Velvet version 1.2.08 [34], with k-mer values ranging from 51 to 71 and a coverage cut-off of 10. MUMmer version 3.0 [35] was used to compare the assembled chloroplast contigs with the closest related complete chloroplast genome sequence available, *Inga leiocalycina* Benth. (Koenen et al. unpublished data). Based on the alignments, contigs were ordered and then merged to produce a single draft genome. Finally, reads were mapped to the assembly using Bowtie 2 [66], and visually inspected for discrepancies using Tablet version 1.13.07.31 [67]. Gaps between contigs were filled by PCR amplification with primers that were designed based on the contig sequences (S3 Table). Reactions were performed in 25 μL reactions using 1X PCR Polymerisation Buffer (Fisher-Biotec, Wembley, Australia), 1.5 mM MgCl_2_, 1.5 μM each forward and reverse primer (GeneWorks; Thebarton, Australia), 0.5 U Taq DNA polymerase (Fisher-Biotec) and 40 ng/μL template DNA. The cycling profile used was: 5 mins at 95°C; followed by 30 secs at 95°C, 45 secs at the annealing temperature (available in S3 Table), and 2 mins at 72°C for 35 cycles; then 4 mins at 72°C.

PCR products were purified prior to sequencing (QIAquick PCR Purification Kit; QIAGEN; Chadstone, Australia), according to the manufacturer’s instructions. Sequencing reactions were performed with forward and reverse primers in separate 10 μL reactions (ABI BigDYE V3.1 Ready-Reaction Kit; Applied Biosystems, USA), following the manufacturer’s directions, and analysed on a 3730XL DNA Analyser (Applied Biosystems). PCR purification and sequencing reactions were performed at the Australian Genome Research Facility (Perth, Australia). Forward and reverse sequences were aligned and manually assessed for incorrect base calls using the CodonCode Aligner software (version 3.7.1; CodonCode Corporation, http://www.codoncode.com/aligner/).

### Genome content

The genome was annotated by comparison with other annotated genomes, particularly from other legumes, using NCBI Blast [68]. All tRNA sequences were also checked against the PlantRNA database [69]. The complete *A*. *ligulata* genome has been deposited into EMBL (accession number: LN555649). GC content for all species was calculated in Geneious (version 6.0.5; created by BioMatters; available from http://www.geneious.com/). The number and location of all tandem repeats greater than 10 bp were detected for all Leguminosae species using the Phobos Tandem Repeat Finder plugin in Geneious. Additionally, the number of forward, reverse, complementary and palindromic repeats were also detected using REPuter [70]. In order to allow comparison between our analysis and previous repeat analyses in legumes [38, 40, 43, 44], we removed one copy of the IR prior to analysis. Repeats greater than 30 bp were then detected using a Hamming distance of 3, corresponding to a sequence identity of over 90%.

### Phylogenetic analyses

Seventy-four protein coding genes were extracted from 21 taxa within the Fabaceae as well as several outgroups, including *Eucalyptus globulus*, *Pyrus pyrifolia*, *Cucumis sativus*, *Morus indica* and *Castanea mollissima*. The *accD* and *ycf4* genes were not used in this analysis due to their absence in *Trifolium subterraneum* and *Pisum sativum*, respectively. All genome sequences were obtained from GenBank (accession numbers in Table 1). Nucleotide sequences were aligned using MAFFT [71] in Geneious. The model of molecular evolution for each gene was determined using the jModelTest [72] function in MetaPiga version 3.1 [73] (models selected can be seen in S4 Table). The alignments from the 74 genes were concatenated and Bayesian inference was performed using MrBayes [74]. Data were analysed with a Gamma model of rate heterogeneity, the proportion of invariable sites was estimated, and for concatenated multilocus datasets, the alignment was partitioned and branch lengths optimised on a per locus basis.

Bayesian analyses were conducted using MrBayes version 3.2 [75] and were run in parallel on the Fornax supercomputer (located at iVEC@UWA) utilising the BEAGLE library [76]. The Fornax computer consists of 96 computer nodes, each with two six-core Intel Xeon X5650CPUs, a NVIDIA Tesla C2075 GPU and 74 GB of memory. Analyses were run for 10 million generations with sampling every 1,000 generation, partitioned datasets and parameter estimation for each partition unlinked. Each analysis consisted of two independent runs, each utilising twelve chains, eleven cold and one hot. Convergence between runs was monitored by finding a plateau in the likelihood score (standard deviation of split frequencies < 0.0015) and the potential scale reduction factor (PSRF) approaching one. Convergence of other parameters within the runs was also checked using Tracer version 1.5.4 [77], with ESS values above 200 for each run. The first 25% of each run was discarded as burn-in for the estimation of consensus topology and the posterior probability for each node. Bayesian run files are available from the authors upon request.

### Assessing *clpP1* divergence

Analysis of selection was performed across the *clpP1* gene using the codeml package in PAML [78]. dN/dS, the ratio of non-synonymous to synonymous nucleotide substitition, was calculated using an alignment of the *clpP1* coding sequences in conjunction with the previously identified phylogeny of *clpP1* (Fig 4). We compared the one-ratio model to a branch-specific model, in which the value of dN/dS was separately estimated for *A*. *ligulata*. A likelihood ratio test was used to evaluate the model of best fit. A model assuming neutral selection (dN/dS fixed to 1) across all branches was also calculated to determine the significance of the *A*. *ligulata* dN/dS value.

### Analysis of *clpP1* RNA

*Acacia ligulata* phyllodes were frozen in liquid nitrogen and ground using a ball mill (Retsch; Haan, Germany). Total RNA was extracted using the QIAGEN RNeasy Plant Mini Kit according to the manufacturer’s instructions (buffer RLC was added to the tissue powder). Contaminating genomic DNA was removed using the TURBO DNA-free kit (Ambion) and the treated RNA was assessed for any remaining genomic DNA contamination by standard PCR (primers A, C, D and F). RNA quantity and quality were assessed using a NanoDrop spectrophotometer (ND-1000), and the Agilent 2100 Bioanalyzer (Agilent, USA), respectively. cDNA was generated from 0.5 μg of total RNA using the Superscript III reverse transcriptase (Invitrogen, Australia) and random primers, according to the manufacturer’s instructions.

PCR primers were designed based on the *A*. *ligulata* DNA sequence in order to test for intron splicing (S5 Table). Reactions were performed in 20 μL volumes using 1X PCR buffer (Invitrogen), 2.5 mM Mg^2+^, 0.2 mM dNTPs (Invitrogen), 0.2 μM forward and reverse primers and Platinum Taq DNA polymerase (Invitrogen). The PCR cycling profile was: 5 mins at 94°C, followed by 30 secs at 94°C, 30 secs at 55°C and 1 min at 72°C for 35 cycles, then 10 mins at 72°C. Multiple products were obtained in one case (lane 4 of Fig 8B). Attempts to improve the stringency of the reaction by designing new primers and adjusting the annealing temp or Mg^++^ concentration were not successful. We obtained the same set of multiple products when pure plasmid containing the 998 bp amplicon was used as a template, so the multiple products are not due to additional copies of the gene elsewhere in the genome. To verify the identification of PCR products generated, products were purified from the gel using the QIAquick Gel Extraction kit (QIAGEN). Purified amplicons were cloned using a pGEM-T Easy vector (Promega, Australia). Plasmid DNA was extracted using the QIAprep Spin Miniprep Kit (QIAGEN), and then sequenced as described above (Macrogen Inc.).

### Search for nuclear *clp* genes

A search database was created from all *A*. *ligulata* reads using the BLAST package version 2.2.10 [68]. The *L*. *luteus* reference was then compared to the *A*. *ligulata* database using blastn. In order to identify nuclear sequences rather than chloroplast sequences, the results were assessed for reads which aligned to the reference across the splice junctions. This analysis was repeated using nucleus-encoded Clp subunits of *M*. *truncatula* and *G*. *max* as reference sequences (Table 2). Potential matches were confirmed by comparing the reads to a database of plant sequences using tblastx and verifying that the best matches were nuclear *CLP* genes.

## Supporting Information

S1 Fig Phylogenetic Reconstruction Using the *ycf4* gene. Phylogenetic reconstruction was performed using MrBayes with a General Time Reversible model with gamma and invariant sites. Posterior probabilities are indicated above the branches.
(EPS)

S2 Fig Multiple Alignment of *clpP1* Coding Sequences. Nucleotide sequences were aligned using MAFFT in Geneious.
(PDF)

S1 Table Repeated Sequences in the Chloroplast Genome of *Acacia ligulata*. The table lists repeated sequences of 30 or more nucleotides in length. The type of repeat (C, complementary; P, palindromic; F, forward; R, reverse) is indicated.
(DOCX)

S2 Table Tandem repeat sequences in the *Acacia ligulata* chloroplast genome. (DOCX)

S3 Table Primers Used to Fill Gaps in the *Acacia ligulata* Chloroplast Genome Sequence. (DOCX)

S4 Table Models, Gamma Distribution and Proportion of Invariant Sites, as Estimated by jModelTest for Each Gene Alignment. (DOCX)

S5 Table Primers Used to Test for *clpP* Intron Splicing in *Acacia ligulata*. (DOCX)
