# Correction: The Complete Sequence of the *Acacia ligulata* Chloroplast Genome Reveals a Highly Divergent *clpP1* Gene

**DOI:** 10.1371/journal.pone.0138367

**Published:** 2015-09-14

**Authors:** Anna V. Williams, Laura M. Boykin, Katharine A. Howell, Paul G. Nevill, Ian Small

The authors would like to amend this article based on the discovery that the originally published *Acacia ligulata* sequence contains assembly errors, which came to light after the publication of the article.

We have since re-sequenced part of the original genome, and in a separate project, sequenced the chloroplast genome of another individual from the same species, using an improved sequencing and assembly protocol. Based on the new results, we can confirm that the original sequence we released contained assembly errors. We have corrected the errors and the European Nucleotide Archive has accepted the revised sequence under the same accession number.

The main differences concern the genome size, which was discovered to be nearly 16kb larger, and the content and structure of repeated sequences; more sequences are repeated than we initially realised. One tRNA gene has been added (encoding tRNA-Ser(GCU)).

To bring the published description fully into line with the revised genome sequence, we hereby replace Figs 1, 2 and 3 and S1 and S2 Tables, and update S3 Table to include the primers used for the additional sequencing that was needed to resolve the assembly problems.

**Fig 1:**
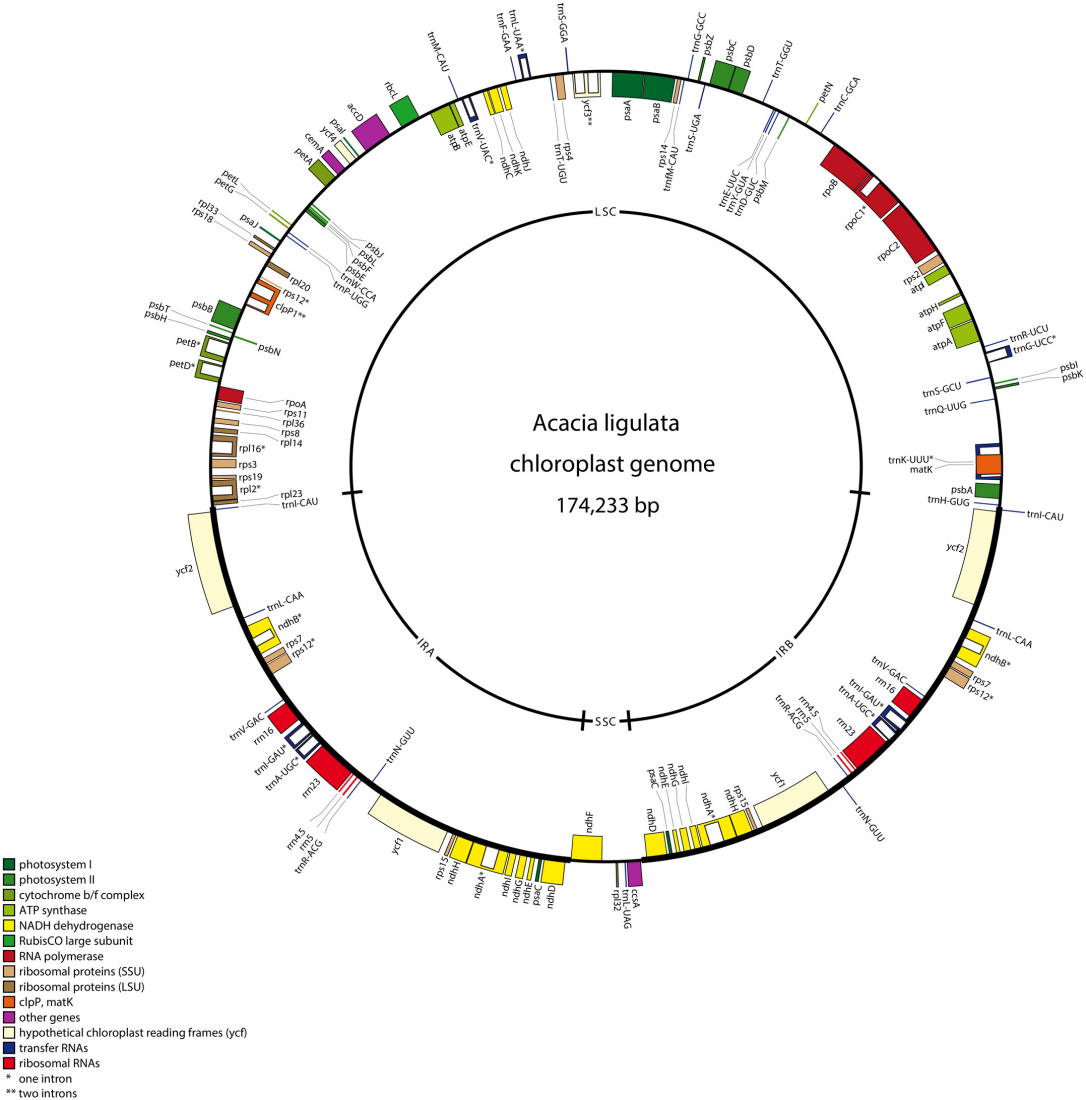
Genome Map of the *Acacia ligulata* Chloroplast. Genes shown on the inside of the circle are transcribed in the clockwise direction and those shown on the outside of the circle are transcribed in the anticlockwise direction. Genes marked with an asterisk contain introns, with the introns indicated by clear boxes. The legend indicates the functional group to which each gene belongs. The figure was generated with OrganelleGenomeDRAW [79].

**Fig 2:**
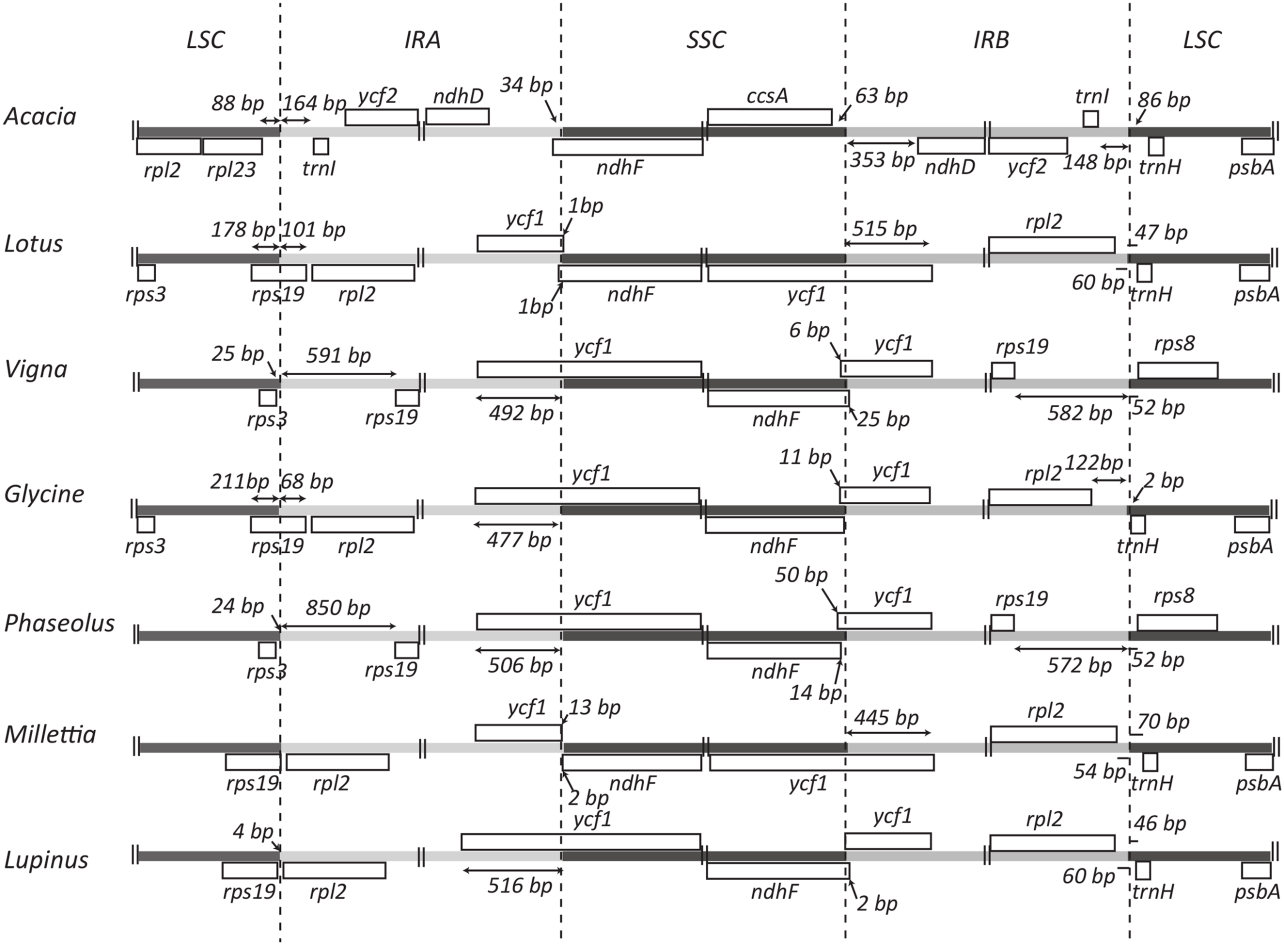
Structure of the LSC/IR junction regions in legume genera. Protein coding regions are indicated by grey boxes with genes below the line being transcribed right to left and those below the line transcribed left to right. The number of base pairs between the end of the gene and the IR is indicated for genes on either side of the junction, unless the junction coincides with the end of a gene.

**Fig 3:**
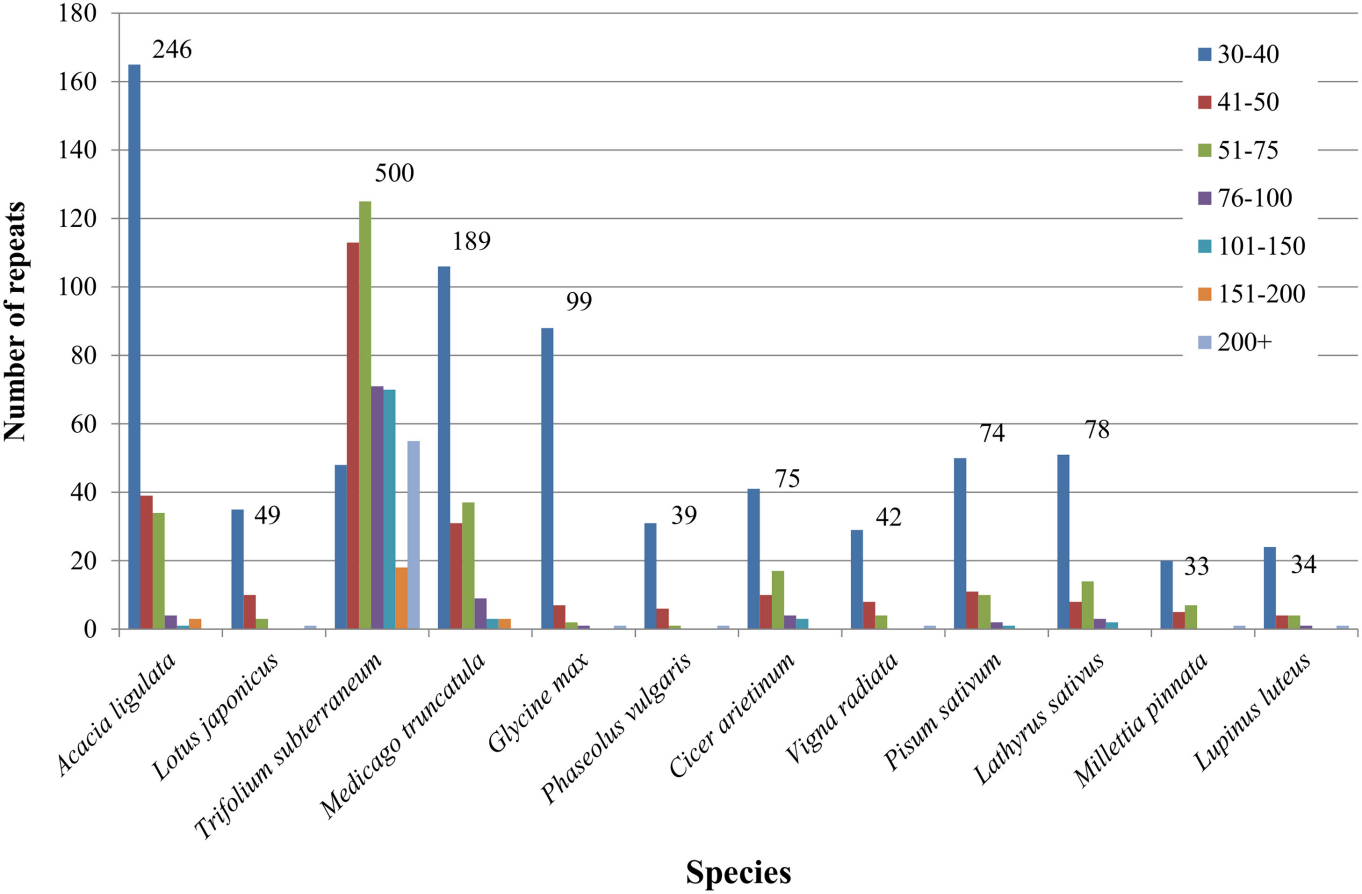
*Acacia ligulata* Chloroplast Genome Repeat Content Compared to that of Other Legume Genomes. Repeats are separated into groups according to their size, and the total number of repeats is shown above the bars.

We have also made alterations to the text concerning the genome size and repeat content:

The fourth sentence of the Abstract should read: “The *A*. *ligulata* chloroplast genome is 174,233 bp in size, comprising inverted repeats of 38,225 bp and single-copy regions of 92,798 bp and 4,985 bp.”

The first three subsections of the Results and Discussion should read:

**Sequencing and assembly**

Dried herbarium material of a specimen of *Acacia ligulata* Benth. was used for DNA extraction. Illumina sequencing of a library prepared from total DNA produced 2,216,882 paired-end reads with a read length of 100 nt. 5.26% of reads were assembled into 23 contigs showing homology to legume plastid DNA. Gaps between contigs were then filled by PCR amplification and Sanger sequencing. The complete assembled chloroplast genome of *A*. *ligulata* is typical in its general structure with a pair of IRs of 38,225 bp, an LSC of 92,798 bp and an SSC of 4,985 bp (Fig 1). Thus, unlike the chloroplast genomes of many of the Papilionoideae, the *A*. *ligulata* genome has inverted repeats and no inversions within the LSC. The total size of the *A*. *ligulata* chloroplast genome is 174,233 bp, 42.8% of which is non-coding DNA. The GC content for the whole genome is 35.4%, while that of the protein-coding, rRNA and tRNA genes is 38.3%, 55.3% and 53.1%, respectively. These values are similar to those in other Leguminosae genomes (see Table 1 for those used in our comparisons).

**Genome content and order**

The *A*. *ligulata* chloroplast genome contains 110 unique genes, including 76 unique protein-coding genes, 4 unique rRNA genes and 30 unique tRNA genes. As is seen throughout the Leguminosae, the *rpl22* gene is absent from the *A*. *ligulata* plastid genome following an ancient transfer to the nuclear genome [33]. The inverted repeat of the *A*. *ligulata* chloroplast genome results in the complete duplication of the *ndhA*, *ndhB*, *ndhD*, *ndhE*, *ndhG*, *ndhH*, *ndhI*, *psaC*, *rps7*, *rps15*, *ycf1* and *ycf2*, as well as exons 1 and 2 of *rps12* and 34 bp of the *ndhF* gene, all four rRNA genes and seven tRNA genes. In contrast to other Leguminosae species that retain their inverted repeat, the IR of the *A*. *ligulata* chloroplast shows complete duplication of the *ycf1* gene. This feature distinguishes *A*. *ligulata* not only from other legumes but also from many other angiosperms, which typically have 1,000 bp or more of the *ycf1* gene included in their IR [38]. Of those legumes that do retain the inverted repeat, that of *A*. *ligulata* is much larger than any of the others (Fig 2).

Eleven protein-coding genes and seven tRNA genes contained at least one intron, with *clpP1*, *rps12* and *ycf3* each containing two introns. This is in contrast to *Cicer arietinum*, *Medicago truncatula*, *Trifolium subterraneum*, *Pisum sativum* and *Lathyrus sativus*, all of which have lost an intron in both *clpP1* and *rps12* [36]. The largest intron was found in *trnK-UUU* (2,544 bp), spanning the entire *matK* gene, whilst *trnL-UAA* contains the smallest intron (543 bp). Two sets of open reading frames overlap: *atpA* and *atpE* overlap by four nucleotides whilst *psbC* and *psbD* overlap by 17 nucleotides, taking the start codon of *psbC* to be the GTG codon at position 36,432, based on the results on *psbC* translation in tobacco [39].

**Repeat content**

The 246 sets of direct and indirect repeats of 30 bp or longer in the *A*. *ligulata* chloroplast genome are listed in S1 Table (not including the large IRs). These include 212 forward repeats, six reverse repeats, four complementary repeats and 24 palindromic repeats. Repeats were found in the *rpl16*, *ndhA*, *ycf3* and *clpP1* introns, and in the *accD*, *psaA* and *psaB* genes. Compared to other legumes, *A*. *ligulata* has a high repeat content. The *Trifolium subterraneum* plastid genome contains by far the greatest number of repeats with 500 repeats in total, while *Millettia pinnata* and *Lupinus luteus* have the fewest, with 33 and 34 repeats, respectively (Fig 3).

The largest repeat in *A*. *ligulata* is a tandem repeat of 195 bp in the *ycf1-trnN* spacer region. The size of this repeat is consistent with those seen in other legumes: for example, some tandem repeats in *Cicer arietinum*, *Medicago truncatula* and *Trifolium subterraneum* are well over 100 bp in length. The *A*. *ligulata* chloroplast genome contains another 48 tandem repeats of 10 bp or more in length (S2 Table). Thirteen were found within genes, including sets in the *ndhA*, *atpF*, *petD* and *clpP1* introns. The remaining repeats were found within intergenic spacer regions. Two sets of tandem repeats observed in *A*. *ligulata* are also found in other legumes: repeat 20 is also in the *rps12-trnV* spacer regions of *Lotus japonicus*, *Millettia pinnata* and *Lupinus luteus*, whereas repeat 27 is also in the *ycf2* genes of *Millettia pinnata* and *Lupinus luteus*.

The sixth sentence of the “Divergence in *clpP1*”subsection should read: “In contrast, the branch leading to *A*. *ligulata* showed a dN/dS ratio (1.09) statistically indistinguishable from that in a model where the dN/dS ratio was fixed as 1 (likelihood ratio test, *P* > 0.99).”

We would also like to expand the Methods section to provide more detailed information on how the assembly was corrected, as follows:

**DNA sequencing**

Dried phyllode material was obtained from a specimen of *Acacia ligulata* Benth. (Fabaceae) held at the Western Australian Herbarium (voucher number: PERTH07807864; collected at Lorna Glen, Western Australia, in 2006). Total genomic DNA was extracted using a CTAB protocol [11]. DNA quality and quantity were assessed using a NanoDrop spectrophotometer (ND-1000; Thermo Fisher Scientific, USA), and agarose gel electrophoresis, respectively. Genome library preparation was performed using a Nextera DNA Sample Preparation Kit (Illumina, San Diego, USA), following the manufacturer’s directions. The library was prepared for sequencing using the cBOT cluster generation system and PE V3 flow cell and cluster chemistry (Illumina). The library was sequenced on a single lane in paired-end mode using the HiSeq2000 platform and V3 SBS kit (Illumina) (100 nt read length). Library preparation and sequencing were both performed at the Ramaciotti Centre for Gene Function Analysis (Sydney, Australia; http://devspace.ddtoo.com/). Total DNA from a second *A*. *ligulata* individual was sequenced following the method above except using the TruSeq DNA Sample Preparation Kit (Illumina) and the MiSeq platform (Illumina) with a read length of 150 nt.

**Genome assembly**

Overlapping paired-end Nextera reads were merged using the software FLASH version 1.2.7 [33] and merged reads were assembled using Velvet version 1.2.08 [34], with k-mer values ranging from 51 to 71 and a coverage cut-off of 10. MUMmer version 3.0 [35] was used to compare the assembled chloroplast contigs with the closest related complete chloroplast genome sequence available, *Inga leiocalycina* Benth. (Koenen et al. unpublished data). Based on the alignments, contigs were ordered and then merged to produce a single draft genome. Gaps between contigs were filled by PCR amplification with primers that were designed based on the contig sequences (S3 Table). Reactions were performed in 25 μL reactions using the 1X PCR Polymerisation Buffer (Fisher-Biotec, Wembley, Australia), 1.5 mM MgCl2, 1.5 μMeach forward and reverse primer (GeneWorks; Thebarton, Australia), 0.5 U Taq DNA polymerase (Fisher-Biotec) and 40 ng/μL template DNA. The cycling profile used was: 5 mins at 95°C; followed by 30 secs at 95°C, 45 secs at the annealing temperature (available in S3 Table), and 2 mins at 72°C for 35 cycles; then 4 mins at 72°C.

PCR products were purified prior to sequencing (QIAquick PCR Purification Kit; QIAGEN; Chadstone, Australia), according to the manufacturer’s instructions. Sequencing reactions were performed with forward and reverse primers in separate 10 μL reactions (ABI BigDYE V3.1 Ready-Reaction Kit; Applied Biosystems, USA), following the manufacturer’s directions, and analysed on a 3730XL DNA Analyser (Applied Biosystems). PCR purification and sequencing reactions were performed at the Australian Genome Research Facility (Perth, Australia). Forward and reverse sequences were aligned and manually assessed for incorrect base calls using the CodonCode Aligner software (version 3.7.1; CodonCode Corporation, http://www.codoncode.com/aligner/).

Subsequent to the initial publication, we were alerted to potential assembly problems. Comparison to the longer read length data from the second individual suggested that two AT-rich repetitive regions had been mis-assembled. These two repetitive regions were amplified by PCR from the original DNA sample and sequenced as described above. The new sequences, together with supporting reads from the original Illumina sequencing run, permitted the correct assembly of these problematic regions. The oligonucleotide primers for these regions have been added to S3 Table.

We would like to thank Donovan Bailey for making us aware of problems in our initial genome assembly.

## Supporting Information

S1 Table Repeated Sequences in the Chloroplast Genome of *Acacia ligulata*. The table lists repeated sequences of 30 or more nucleotides in length. The type of repeat (C, complementary; P, palindromic; F, forward; R, reverse) is indicated.
(DOCX)

S2 Table Tandem repeat sequences in the *Acacia ligulata* chloroplast genome. (DOCX)

S3 Table Primers Used to Fill Gaps in the *Acacia ligulata* Chloroplast Genome Sequence. (DOCX)
