# Supplementary material for: The Complete Sequence of the Acacia ligulata Chloroplast Genome Reveals a Highly Divergent clpP1 Gene
Source: PLoS One. 2015 May 8;10(5):e0125768. doi: 10.1371/journal.pone.0125768 (PMC4425659; doi:10.1371/journal.pone.0125768)
Supplement: S2 Fig — Nucleotide sequences were aligned using MAFFT in Geneious. (PDF) [file pone.0125768.s002.pdf]

```
                                           10        20        30        40        50        60
                                            |         |         |         |         |         |
KC893635_GLYCINE_CANESCENS      ATGCCCATTGGTGTTCCAAGAGTACCCTTTCGAAGCCCTGGGGAAGAAGATGCATCTTGG
KC893637_GLYCINE_FALCATA        ATGCCCATTGGTGTTCCAAGAGTACCCTTTCGAAGCCCTGGGGAAGAAGATGCATCTTGG
KC893633_GLYCINE_TOMENTELLA     ATGCCCATTGGTGTTCCAAGAGTACCCTTTCGAAGCCCTGGGGAAGAAGATGCATCTTGG
KC893638_GLYCINE_SYNDETIKA      ATGCCCATTGGTGTTCCAAGAGTACCCTTTCGAAGCCCTGGGGAAGAAGATGCATCTTGG
KC893636_GLYCINE_DOLICHOCARPA   ATGCCCATTGGTGTTCCAAGAGTACCCTTTCGAAGCCCTGGGGAAGAAGATGCATCTTGG
KC893634_GLYCINE_STENOPHITA     ATGCCCATTGGTGTTCCAAGAGTACCCTTTCGAAGCCCTGGGGAAGAAGATGCATCTTGG
KC893632_GLYCINE_CYRTOLOBA      ATGCCCATTGGTGTTCCAAGAGTACCCTTTCGAAGCCCTGGGGACGAAGATGCATCTTGG
KF611800_GLYCINE_SOJA           ATGCCCATTGGTGTTCCAAGAGTACCCTTTCGAAGCCCTGGGGAAGAAGATGCATCTTGG
NC_007942_GLYCINE_MAX           ATGCCCATTGGTGTTCCAAGAGTACCCTTTCGAAGCCCTGGGGAAGAAGATGCATCTTGG
JQ755301_VIGNA_UNGUICULATA      ATGCCCATTGGTGTTCCAAGAGTACCCTTTCGAACCCCCGGGGATAAAGATGCATCTTGG
NC_013843_VIGNA_RADIATA         ATGCCCATTGGTGTTCCAAGAGTACCCTTTCGAATCCCTGGGGATAGAGATGCAGCTTGG
AP012598_VIGNA_ANGULARIS        ATGCCCATTGGTGTTCCAAGAGTACCCTTTCGACTCCCCGGGGATAGAGATGCAGTTTGG
NC_009259_PHASEOLUS_VULGARIS    ATGCCCATTGGTGTTCCAAGAGTACCCTTTCGAACCCCCGGGGACCGTGATGCATCTTGG
NC_016708_MILLETTIA_PINNATA     ATGCCCATTGGTGTTCCAAAAGTATCCTTCCGAACTCCCGGAGATGGAGATGCATCTTGG
NC_014057_PISUM_SATIVUM         ATGCCAGTAGGTGTTCCAAAGGTAGGCTTTGAAGTTCCTGGCGATGATGAAGCTTCTTGG
NC_014063_LATHYRUS_SATIVUS      ATGCCAGTGGGTGTTCCAAAGGTAGCCTTTCAAATTCCTGGCGAGGATGAAGCTACTTGG
EU849487_TRIFOLIUM_SUBTERRANEUM ATGCCGGTTGGAGTTCCCAAGGTCCCCTATTTAATGCCGGAGGAAGATGAGGCATCTTGG
NC_011163_CICER_ARIETINUM       ATGCCACTTGGTGTACCAAAGGTACCTTTTCTCGTTCCTGGAGATGATGAGGCATCTTGG
NC_003119_MEDICAGO_TRUNCATULA   ATGCCAATTGGTGTTCCAAAAGTACCCTTTCAACTTCCTGAAGAAGATGAGGCATCTTGG
DQ226511_MORUS_INDICA           ATGCCTATTGGTGTTCCAAAAGTACCTTTTCGAAGTCCTGGAGACGAAGATGCATCGTGG
NC_015996_PYRUS_PYRIFOLIA       ATGCCTATTGGTGTTCCAAGAGTACCTTTTCGAAGTCCTGGAGAGGAAGATGCATTGTGG
NC_014674_CASTANEA_MOLLISSIMA   ATGCCTATTGGTGTTCCAAAAGTACCTTTTCGAAGTCCTGGAGAGGAAGATGCATCGTGG
KC180787_EUCALYPTUS_GLOBULUS    ATGCCTATTGGTGTTCCAAAAGTACCCTTTCGAAGTCCTGGAGAGGAAGATGCCTCTTGG
DQ119058_CUCUMIS_SATIVUS        ATGCCTGTTGGTGTTCCAAAAGTACCCTTTCGACTTCCCGGAGAAGAAGATGCATCGTGG
ACACIA_LIGULATA                 ATGCCTTTTGGTGTTCCAAAGGTACCTTATCAAGGTCCTGAAGATGAAAACGCGTCTTGG
NC_023090_LUPINUS_LUTEUS        ATGCCCATTGGTGTTCCAAAAGTACCCTTTCGAAGTCCTGGAGAAGAAGATGCATCTTGG
AP002983_LOTUS_JAPONICUS        ATGCCCATTGGTGTTCCAAAAGTGCCCTTTCGAAGTCCTGGAGAGGAAGATGCATCTTGG

                                           70        80        90       100       110       120
                                            |         |         |         |         |         |
KC893635_GLYCINE_CANESCENS      GTTGATATATACAATCGACTTTATCGAGAAAGATTACTTTTTTTAGGTCAAGAGGTTGAT
KC893637_GLYCINE_FALCATA        GTTGATATATACAATCGACTTTATCGAGAAAGATTACTTTTTTTAGGTCAAGAGGTTGAT
KC893633_GLYCINE_TOMENTELLA     GTTGATATATACAATCGACTTTATCGAGAAAGATTACTTTTTTTAGGTCAAGAGGTTGAT
KC893638_GLYCINE_SYNDETIKA      GTTGATATATACAATCGACTTTATCGAGAAAGATTACTTTTTTTAGGTCAAGAGGTTGAT
KC893636_GLYCINE_DOLICHOCARPA   GTTGATATATACAATCGACTTTATCGAGAAAGATTACTTTTTTTAGGTCAAGAGGTTGAT
KC893634_GLYCINE_STENOPHITA     GTTGATATATACAATCGACTTTATCGAGAAAGATTACTTTTTTTAGGTCAAGAGGTTGAT
KC893632_GLYCINE_CYRTOLOBA      GTTGATATATACAATCGACTTTATCGAGAAAGATTACTTTTTTTAGGTCAAGAGGTTGAT
KF611800_GLYCINE_SOJA           GTTGATATATACAATCGACTTTATCGAGAAAGATTACTTTTTTTAGGTCAAGAGGTTGAT
NC_007942_GLYCINE_MAX           GTTGATATATACAATCGACTTTATCGAGAAAGATTACTTTTTTTAGGTCAAGAGGTTGAT
JQ755301_VIGNA_UNGUICULATA      GTTGATATATACAATCGACTTTATCGAGAAAGATTACTTTTTTTAGGTCAAGATGTTGAT
NC_013843_VIGNA_RADIATA         ATTGATATATACAATCGACTTTATCGAGAAAGATTACTTTTTTTAGGTCAAGATGTTGAT
AP012598_VIGNA_ANGULARIS        ATTGATATATACAATCGACTTTATCGAGAAAGATTACTTTTTTTAGGTCAAGATGTTGAT
NC_009259_PHASEOLUS_VULGARIS    GTTGATATATACAATCGACTTTATCGAGAAAGATTACTTTTTTTAGGTCAAGACGTTGAT
NC_016708_MILLETTIA_PINNATA     GTTGATATATACAATCGACTTTATCGAGAAAGATTACTTTTTTTAGGCCAAGAGGTTGAT
NC_014057_PISUM_SATIVUM         ATTGACTTATACCATCAACTTTTTTTCGACAGACTACTTTTTTTAGGTCAAGAGGTTGAG
NC_014063_LATHYRUS_SATIVUS      GTTGACTTATACCATCAACTTTTTTACAGCAGACTTCTTTTTTTAGGTGACGAGATTGAG
EU849487_TRIFOLIUM_SUBTERRANEUM GTTGATTTATACCATCTTCTTTACGAAGAAAGACTTTTATTTTTAGGGCAAGAAGTAAAC
NC_011163_CICER_ARIETINUM       ATTGACTTATACAATCGACTTTTTCAAGAAAGACTACTTTTTTTAGGTCAAGAAGTAAAC
NC_003119_MEDICAGO_TRUNCATULA   GTTGATTTATACAATCGACTTTTTCAAGAAAGATTACTTTTTTTAGGTCAAGAGGTCAAC
DQ226511_MORUS_INDICA           GTTGACGTATACAACCGACTTTATCGAGAAAGATTACTTTTTTTAGGCCAAGAGGTTGAT
NC_015996_PYRUS_PYRIFOLIA       ATTGACATATACAACCGACTTTATCGAGAACGATTACTTTTTTTAGGCCAAGGGGTTGAT
NC_014674_CASTANEA_MOLLISSIMA   GTTGACATATACAACCGACTTTATCGAGCAAGATTACTTTTTTTAGGCCAAGAGGTTGAT
KC180787_EUCALYPTUS_GLOBULUS    GTTGACGTATACAACCGACTTTATCGAGAAAGATTACTTTTTTTAGGCCAAGAGGTTGAT
DQ119058_CUCUMIS_SATIVUS        GTTGACCTATACAACCGACTTTATCGACAAAGATTACTTTTTTTAGGCCAAGATGTTAAT
ACACIA_LIGULATA                 TTTGACTTATACAACGGACTTTATAAAACAAGAGCCGTTTTTATAGGCCGTAAGCTTGAT
NC_023090_LUPINUS_LUTEUS        GTTGACATATACAACCGACTTTATCGCGAAAGACTACTTTTTTTAGGCCAAGAGGTTGAT
AP002983_LOTUS_JAPONICUS        GTTGACATATACAATCGACTTTATAGAGAAAGACTGCTTTTTTTAGGCCAGGAAGTTAAT

                                          130       140       150       160       170       180
                                            |         |         |         |         |         |
KC893635_GLYCINE_CANESCENS      AGTGAAATATCGAATCAACTAATTAGTCTTATGGTATATCTTAGTATAGAGGAA---GAG
KC893637_GLYCINE_FALCATA        AGTGAAATATCGAATCAACTAATTAGTCTTATGGTATATCTTAGTATAGAGGAA---GAG
KC893633_GLYCINE_TOMENTELLA     AGTGAAATATCGAATCAACTAATTAGTCTTATGGTATATCTTAGTATAGAGGAA---GAG
KC893638_GLYCINE_SYNDETIKA      AGTGAAATATCGAATCAACTAATTAGTCTTATGGTATATCTTAGTATAGAGGAA---GAG
KC893636_GLYCINE_DOLICHOCARPA   AGTGAAATATCGAATCAACTAATTAGTCTTATGGTATATCTTAGTATAGAGGAA---GAG
KC893634_GLYCINE_STENOPHITA     AGTGAAATATCGAATCAACTAATTAGTCTTATGGTCTATCTTAGTATAGAGGAA---GAG
KC893632_GLYCINE_CYRTOLOBA      AGTGAAATATCGAATCAACTAATTAGTCTTATGGTATATCTTAGTATAGAGGAA---GAG
KF611800_GLYCINE_SOJA           AGTGAAATATCGAATCAACTAATTAGTCTTATGGTATATCTTAGTATAGAAGAA---GAG
NC_007942_GLYCINE_MAX           AGTGAAATATCGAATCAACTAATTAGTCTTATGGTATATCTTAGTATAGAAGAA---GAG
JQ755301_VIGNA_UNGUICULATA      AGTGAAATATCGAATCAACTTATTAGTATTATGATATATCTGAGCATAGAGAAA---GAG
NC_013843_VIGNA_RADIATA         AGTGAAATATCGAATCAACTTATTAGTATTATGATCTATCTGAGCATAGAGAAA---GAG
AP012598_VIGNA_ANGULARIS        AGTGAAATATCGAATCAACTTATTAGTCTTATGATCTATCTGAGCATAGAGAAA---GAG
NC_009259_PHASEOLUS_VULGARIS    AGTGAAATATCGAATCAACTTATTAGTCTTATGATATATCTGAGCATAGAGAAA---GAG
NC_016708_MILLETTIA_PINNATA     AGTCAACTATCGAATCAACTTATTGGTATTATGCTGTATCTCAGTATAGAGAGC---GAT
NC_014057_PISUM_SATIVUM         AGTGAAATATCAAATCAAGTTTGTGGTATGATGATATATCTCAGTTTAGAGAAC---AAG
```

```
NC_014063_LATHYRUS_SATIVUS      AGTGAACTAGCAAATCAAGTTGCTGGTATGATGATATATCTCAGTTTAGAGAAC---AAG
EU849487_TRIFOLIUM_SUBTERRANEUM AGTGAAATTTCAAATCAATTGAATGGTCTTATGGTATATCTTAGTTTACAGGAC---AAG
NC_011163_CICER_ARIETINUM       AGTGAAATATCAAACCAACTTGTTGGTCTAATGGTATATCTAAGTTTAGAGGAC---AAG
NC_003119_MEDICAGO_TRUNCATULA   ACTGAAATATCAAATCAACTTGTTGGTCTAATGGTATATCTCAGTTTAGAGGAC---AAG
DQ226511_MORUS_INDICA           AGCGAGATCTCGAATCAACTTATTGGTCTTATGGTATTTCTCAGTATAGAGGAT---GAT
NC_015996_PYRUS_PYRIFOLIA       AGCGAGATCTCGAATCAACTTATTGGTCTTATGGTATATCTCAGTATAGAGGAT---GAT
NC_014674_CASTANEA_MOLLISSIMA   AGCGATCTCTCGAATCAAATTATTGGTCTTATGGTATATCTCAGTCTAGAGGAT---GAT
KC180787_EUCALYPTUS_GLOBULUS    AGCGAGATTTCGAATCAACTTATTGGTCTTATGGTATATCTCAGTATAGAGAAT---GAT
DQ119058_CUCUMIS_SATIVUS        AATGAGATCTCGAATCAAATTATGGGTCTTATGGTATATCTCAGTATAGAGGAT---GGT
ACACIA_LIGULATA                 GATGAACTAATGAATCAAACTGTGGGAATTTTGGTGCATCTCCATCTAAACAATCCTGAC
NC_023090_LUPINUS_LUTEUS        GGTGAAATATCAAATCAACTTATCGGCCTTATGGTATATCTCAGTATAGAGGAC---GAT
AP002983_LOTUS_JAPONICUS        AGTGAAATATCAAATCAACTTATTGGTCTTATGGTATATCTCAGTATAGAGGAT---GAT

                                        190       200       210       220       230       240
                                         |         |         |         |         |         |
KC893635_GLYCINE_CANESCENS      AATAAAGATTTGTATCTGTTTATAAATTCTCCGGGGGGGTGGGTAATACCCGGAATAGCT
KC893637_GLYCINE_FALCATA        AATAAGGATTTGTATCTGTTTATAAATTCTCCGGGGGGGTGGGTAATACCCGGAATAGCT
KC893633_GLYCINE_TOMENTELLA     AATAAAGATTTGTATCTGTTTATAAATTCTCCGGGGGGGTGGGTAATACCCGGAATAGCT
KC893638_GLYCINE_SYNDETIKA      AATAAAGATTTGTATCTGTTTATAAATTCTCCGGGGGGGTGGGTAATACCCGGAATAGCT
KC893636_GLYCINE_DOLICHOCARPA   AATAAAGATTTGTATCTGTTTATAAATTCTCCGGGGGGGTGGGTAATACCCGGAATAGCT
KC893634_GLYCINE_STENOPHITA     AATAAAGATTTGTATCTGTTTATAAATTCTCCGGGTGGGTGGGTAATACCCGGAATAGCT
KC893632_GLYCINE_CYRTOLOBA      AATAAAGATTTGTATTTGTTTATAAATTCTCCGGGGGGGTGGGTAATACCCGGAATAGCT
KF611800_GLYCINE_SOJA           AATAAAGATTTGTATCTGTTTATAAATTCTCCGGGGGGGTGGGTAATACCCGGAATAGCT
NC_007942_GLYCINE_MAX           AATAAAGATTTGTATCTGTTTATAAATTCTCCGGGGGGGTGGGTAATACCCGGAATAGCT
JQ755301_VIGNA_UNGUICULATA      AATAAAGAATTGTCTCTGTTTATAAACTCTCCGGGGGGGTGGGTAATACCTGGAATAGCT
NC_013843_VIGNA_RADIATA         AATAAAGAATTGTCTCTGTTTATAAACTCTCCGGGGGGGTGGGTAATACCTGGAATAGCT
AP012598_VIGNA_ANGULARIS        AATAAAGAATTGTCTCTGTTTATAAACTCTCCGGGGGGGTGGGTAATACCTGGAATAGCT
NC_009259_PHASEOLUS_VULGARIS    AATAAAGATTTATATCTGTTTATAAACTCTCCGGGCGGTTGGGTAATACCTGGAATAGCT
NC_016708_MILLETTIA_PINNATA     AACAAAGATTTGTGTCTGTTTGTAAATTCTCCGGGTGGGTGGGTAATACCTGGACTAGCT
NC_014057_PISUM_SATIVUM         TACAAAAATTTGTATCTGTTTATAAAATCTCCAGGCGGAGAGGTTCTATCTGGATTGGCC
NC_014063_LATHYRUS_SATIVUS      AACAAAGATTTGTATCTGTTTATAAATTCTCCCGGCGGAGAGGTTATGTCTGGAATGGCC
EU849487_TRIFOLIUM_SUBTERRANEUM ACAAAAGATTTGTATCTGTTTATAAATTCTCCGGGCGGAGAGCTAATGTCCGGAATGGCT
NC_011163_CICER_ARIETINUM       AACAAAGATTTGTATTTGTTTATAAATTCTCCGGGCGGAGAGGTAATATCTGGAATGGCA
NC_003119_MEDICAGO_TRUNCATULA   ACCAGAGATTTGTATTTGTTTATAAATTCTCCGGGCGGAGAGGTAATATCTGGAATGGCT
DQ226511_MORUS_INDICA           ACTAAGGATCTCTATTTGTTTATAAACTCTCCCGGCGGATGGGTAATACCTGGATTAGGT
NC_015996_PYRUS_PYRIFOLIA       ACCAAAGATCTATATTTGTTTATAAATTCTCCTGGCGGATGGGTAATACCCGGAGTAGCT
NC_014674_CASTANEA_MOLLISSIMA   AGCAAAGATCTGTATTTGTTTATAAACTCTCCCGGGGGGTGGGTAATACCCGGAGTAGCT
KC180787_EUCALYPTUS_GLOBULUS    AACAAGGATCTGTATTTGTTTATAAACTCTCCTGGCGGATGGGTAATCCCCGGAGTAGCT
DQ119058_CUCUMIS_SATIVUS        ACCAAAGATCAGTATTTATTTATAAACTCTCCCGGTGGATCGGTAATACCCGGAGTAGGT
ACACIA_LIGULATA                 AAAAAAGACATCCAAGTTTTTATAAATTGTCCAGGTGGATGGATGGTATCCGGAATAGCT
NC_023090_LUPINUS_LUTEUS        AACCAAGATTTGTATCTGTTTATAAATTCCCCAGGCGGATGGGTAATCCCCGGAATAGCT
AP002983_LOTUS_JAPONICUS        AAGAAAGATTTGTATCTGTTTATTAATTCTCCGGGAGGATGGGTAATCCCCGGAATAGCT

                                        250       260       270       280       290       300
                                         |         |         |         |         |         |
KC893635_GLYCINE_CANESCENS      ATTTATGATACTATGCAATTTGTACAACCAGATGTACAGACAGTATGTATGGGATTAGCC
KC893637_GLYCINE_FALCATA        ATTTATGATACTATGCAATTTGTACAACCAGATGTACAGACAGTATGTATGGGATTAGCC
KC893633_GLYCINE_TOMENTELLA     ATTTATGATACTATGCAATTTGTACAACCAGATGTACAGACAGTATGTATGGGATTAGCC
KC893638_GLYCINE_SYNDETIKA      ATTTATGATACTATGCAATTTGTACAACCAGATGTACAGACAGTATGTATGGGATTAGCC
KC893636_GLYCINE_DOLICHOCARPA   ATTTATGATACTATGCAATTTGTACAACCAGATGTACAGACAGTATGTATGGGATTAGCC
KC893634_GLYCINE_STENOPHITA     ATTTATGATACTATGCAATTTGTACAACCAGATGTACAAACAGTATGTATGGGATTAGCC
KC893632_GLYCINE_CYRTOLOBA      ATTTATGATACTATGCAATTTGTACAACCAGATGTACAAACAGTATGTATGGGATTAGCC
KF611800_GLYCINE_SOJA           ATTTATGATACTATGCAATTTGTACAACCAGATGTACAGACAGTATGTATGGGATTAGCC
NC_007942_GLYCINE_MAX           ATTTATGATACTATGCAATTTGTACAACCAGATGTACAGACAGTATGTATGGGATTAGCC
JQ755301_VIGNA_UNGUICULATA      ATTTATGATACTATGCAATTTGTACAACCAGATGTACAGACAGTATGTCTGGGATTAGCT
NC_013843_VIGNA_RADIATA         ATTTATGATACTATGCAATTTGTACAACCAGATGTACAGACAGTATGTCTGGGATTAGCT
AP012598_VIGNA_ANGULARIS        ATTTATGATACTATGCAATTTGTACAACCAGATGTACAGACAGTATGTCTGGGATTAGCT
NC_009259_PHASEOLUS_VULGARIS    CTTTATGATACTATGCAATTTGTACAACCAGATGTACAGACAGTATGTTTGGGATTAGCT
NC_016708_MILLETTIA_PINNATA     ATTTTTGATACTATGCAATACATAGAACCAGACGTACAGACAGTATGTATGGGATTAGCC
NC_014057_PISUM_SATIVUM         ATTTTTGATATTATGCAATTTGTGGAAGCAGAAGTACAGACAGTATGCGTAGGATTAGCC
NC_014063_LATHYRUS_SATIVUS      ATTTTTGATACTATGCAAGTTGTAGAAGCACAAGTACATACAATATGCATGGGATTAGCC
EU849487_TRIFOLIUM_SUBTERRANEUM ATGTTTGATACTATGCAATTTGTAAAAGCCGAAGTACAGACAGTATGCGTAGGAATAGCC
NC_011163_CICER_ARIETINUM       ATATTTGATACTATGCAATTTGTAGAAGCAGAAGTACAGACAGTATGCGTAGGATTAGCC
NC_003119_MEDICAGO_TRUNCATULA   ATATTTGATATTATGCAAGTTGTAGAAGCAGAAGTACATACAGTATGCGTAGGACTAGCC
DQ226511_MORUS_INDICA           ATTTATGATACTATGCAATTTGTGCAACCAGACGTACAAACAATATGCATGGGATTAGCC
NC_015996_PYRUS_PYRIFOLIA       ATTTATGATACTATGCAATTTGTGCGACCAGATATACAGACAATATGCATGGGATTAGCC
NC_014674_CASTANEA_MOLLISSIMA   ATTTATGATACTATGCAATTTGTACAACCAGATGTACAGACAATATGCATGGGATTGGCC
KC180787_EUCALYPTUS_GLOBULUS    ATTTATGATACTATGCAATTTGTGCAACCCGATGTGCATACAATATGCATGGGATTAGCC
DQ119058_CUCUMIS_SATIVUS        CTTTTTGATACTATGCAATTTGTGTCACCAGATGTCCATACAATATGTATTGGATTAGCC
ACACIA_LIGULATA                 ATTGCTGATTTGATGGAAACAGTGCAACCAGATGTACAGACAATATGCGTGGGAACAGCC
NC_023090_LUPINUS_LUTEUS        ATTTATGATACTATGCAATTTGTACAACCAGATGTACAAACAGTATGCATGGGATTAGCT
AP002983_LOTUS_JAPONICUS        ATTTATGATACTATGCAATTTGTACAACCGGATGTACAGACAGTATGCATGGGATTAGCC

                                        310       320       330       340       350       360
                                         |         |         |         |         |         |
KC893635_GLYCINE_CANESCENS      GCTTCAATGGGATCCTTTCTTTTGGCAGGAGGAGAAATTACCAAACGTTTAGCATTCCCT
KC893637_GLYCINE_FALCATA        GCTTCAATGGGATCCTTTCTTTTGGCAGGAGGAGAAATTACCAAACGTTTAGCATTCCCT
```

```
KC893633_GLYCINE_TOMENTELLA      GCTTCAATGGGATCCTTTCTTTTGGCAGGAGGAGAAATTACCAAACGTTTAGCATTCCCT
KC893638_GLYCINE_SYNDETIKA       GCTTCAATGGGATCCTTTCTTTTGGCAGGAGGAGAAATTACCAAACGTTTAGCATTCCCT
KC893636_GLYCINE_DOLICHOCARPA    GCTTCAATGGGATCCTTTCTTTTGGCAGGAGGAGAAATTACCAAACGTTTAGCATTCCCT
KC893634_GLYCINE_STENOPHITA      GCTTCAATGGGATCCTTTCTTTTGGCAGGAGGAGAAATTACCAAACGTTTAGCATTCCCT
KC893632_GLYCINE_CYRTOLOBA       GCTTCAATGGGATCCTTTCTTTTGGCAGGAGGAGAAATTACCAAACGTTTAGCATTCCCT
KF611800_GLYCINE_SOJA            GCTTCAATGGGATCCTTTCTTTTGGCAGGAGGAGAAATTACCAAACGTTTAGCATTCCCT
NC_007942_GLYCINE_MAX            GCTTCAATGGGATCCTTTCTTTTGGCAGGAGGAGAAATTACCAAACGTTTAGCATTCCCT
JQ755301_VIGNA_UNGUICULATA       GCTTCAATGGGATCCTTTCTTTTGGCAGGAGGCACAATTACCAAACGTCTTGCATTTCCT
NC_013843_VIGNA_RADIATA          GCTTCAATGGGATCCTTTCTTTTGGCAGGAGGCACAATTACCAAACGTCTTGCATTTCCT
AP012598_VIGNA_ANGULARIS         GCTTCAATGGGATCCTTTCTTTTGGCAGGAGGCACAATTACCAAACGTCTTGCATTTCCT
NC_009259_PHASEOLUS_VULGARIS     GCTTCAATGGGATCCTTTCTTTTGGCAGGAGGCACAATTACCAAACGTCTTGCATTTCCT
NC_016708_MILLETTIA_PINNATA      GCTTCAATGGGATCCTTTCTTTTGGCAGCCGGAGAAATTACCAAACGTCTAGCATTCCCT
NC_014057_PISUM_SATIVUM          GCTTCAATGGCATCTTTGATTTTGCTAGGAGGCGAAATTACCAAACGTCTAGCATTCCCT
NC_014063_LATHYRUS_SATIVUS       GCTTCAATGGCATCTCTTATTTTGGCAGGAGGCGGAATTACCAAACGTCTAGCATTTCCT
EU849487_TRIFOLIUM_SUBTERRANEUM  GTTTCAATGGCATCCCTTCTTTTGCTAGGGGGAGAAATTACCAAACGTCTAGCATTTTCT
NC_011163_CICER_ARIETINUM        GCTTCAATGGGATCGCTTATTTTGGTAGGAGGAGAAATTACCAAACGTCTAGCATTCCCT
NC_003119_MEDICAGO_TRUNCATULA     GCTTCAATGGGATCTCTTCTTTTGGTCGGAGGGGAATTTACCAAACGTCTAGCATTCCCT
DQ226511_MORUS_INDICA            GCTTCAATGGGATCTTTTATTCTGGTCGGAGGAGAAATTACCAAACGTCTAGCATTCCCT
NC_015996_PYRUS_PYRIFOLIA        GCTTCAATGGGATCTTTTATTCTGGTCGGAGGAGAAATTACCAAACGTCTAGCATTCCCT
NC_014674_CASTANEA_MOLLISSIMA    GCTTCAATAGGATCTTTTCTTCTGGTCGGGGGAGAAATTACCAAACGTCTAGCATTCCCT
KC180787_EUCALYPTUS_GLOBULUS     GCTTCAATGGGATCTTTTGTCCTGGTCGGCGGAGAGATTACCAAACGTCTAGCATTCCCT
DQ119058_CUCUMIS_SATIVUS         GCTTCAATGGGATCTTTTATTCTAGTCGGAGGAGAAATTACCAAACGTCTAGCATTCCCT
ACACIA_LIGULATA                  GCTTCAATGGCAACTTTTATTCTCCTATCAGGAGAACCGACAAAATGTACAGCATTCCCT
NC_023090_LUPINUS_LUTEUS         GCTTCAATGGGATCCTTTATTTTGGCAGGAGGCGAAATTACCAAACGTCTAGCATTTCCT
AP002983_LOTUS_JAPONICUS         GCTTCAATGGGATCTTTTGTTTTGGCAGGCGGAAAAATTACCAAACGTCTAGCATTCCCT

                                         370       380       390       400       410       420
                                           |         |         |         |         |         |
KC893635_GLYCINE_CANESCENS       CACGCTAGGGTAATGATCCACCAACCCGC---TAGTTCTTTTTATGAGGCACAAACGGGA
KC893637_GLYCINE_FALCATA         CACGCTAGGGTAATGATCCACCAACCCGC---TAGTTCTTTTTATGAGGCACAAACGGGA
KC893633_GLYCINE_TOMENTELLA      CACGCTAGGGTAATGATCCACCAACCCGC---TAGTTCTTTTTATGAGGCACAAACGGGA
KC893638_GLYCINE_SYNDETIKA       CACGCTAGGGTAATGATCCACCAACCCGC---TAGTTCTTTTTATGAGGCACAAACGGGA
KC893636_GLYCINE_DOLICHOCARPA    CACGCTAGGGTAATGATCCACCAACCCGC---TAGTTCTTTTTATGAGGCACAAACGGGA
KC893634_GLYCINE_STENOPHITA      CACGCTAGGGTAATGATCCACCAACCCGC---TAGTTCTTTTTATGAGGCACAAACGGGA
KC893632_GLYCINE_CYRTOLOBA       CACGCTAGGGTAATGATACACCAACCCGC---TAGTTCTTTTTATGAGGCACAAACGGGA
KF611800_GLYCINE_SOJA            CACGCTAGGGTAATGATCCACCAACCCGC---TAGTTCTTTTTATGAGGCACAAACGGGA
NC_007942_GLYCINE_MAX            CACGCTAGGGTAATGATCCACCAACCCGC---TAGTTCTTTTTATGAGGCACAAACGGGA
JQ755301_VIGNA_UNGUICULATA       CACGCTAGGGTAATGATCCACCAACCTGC---TAGTTCTTTTTATGAGGCACAAGCGGGA
NC_013843_VIGNA_RADIATA          CACGCTAGGGTAATGATCCACCAACCTGC---TAGTTCTTTTTATGAGGCACAAGCGGGA
AP012598_VIGNA_ANGULARIS         CACGCTAGGGTAATGATCCACCAACCTGC---TAGTTCTTTTTATGAGGCACAAGCGGGA
NC_009259_PHASEOLUS_VULGARIS     CACGCTAGGGTAATGATCCACCAACCCGC---TAGTTCTTTTTATGAAGCACAAGCGGGA
NC_016708_MILLETTIA_PINNATA      CACGCTAGGGTAATGATCCACCAACCCGC---TAGTTCTTTTTATGAAGCACAAACGGGA
NC_014057_PISUM_SATIVUM          CACGCTAGGGTAATGATCCATCAACCTGC---TAGTTCTGCTTATGATGGACAATCGGGA
NC_014063_LATHYRUS_SATIVUS       CACGCTAGGGTAATGATCCATCAACCTCA---TAGTGCCCCTTATGAGGGACCATCAGGA
EU849487_TRIFOLIUM_SUBTERRANEUM  CACGCTAGGGTCATGATGCATGAACCTGC---TAGTGTGTTTGAAGACGATAAAACAGGC
NC_011163_CICER_ARIETINUM        CACGCTAGGGTAATGATCCATCAACCTGC---TACTTCTTTTTATGAGGGACAAACAGTA
NC_003119_MEDICAGO_TRUNCATULA     CACGCTAGGGTAATGATCCATCAACCTGC---TACTTCTTTGTATGAGGGACAAGTAGGA
DQ226511_MORUS_INDICA            CACGCTAGGGTAATGATCCATCAACCTGC---TAGTTCTTTTTATGAGGCACAGACGGGA
NC_015996_PYRUS_PYRIFOLIA        CACGCTAGGGTAATGATCCATCAACCTGC---TAGTTCTTTTTATGAGGCACAGACGGGA
NC_014674_CASTANEA_MOLLISSIMA    CATGCTAGGGTAATGATCCATCAACCTGC---TAGTTCTTTTTATGAGGCACCCGCAGGA
KC180787_EUCALYPTUS_GLOBULUS     CACGCTAGGGTAATGATACATCAACCTGC---GAGTTCTTTTTATGAGGCACAAACGGGA
DQ119058_CUCUMIS_SATIVUS         CACGCTAGGGTAATGATCCATCAACCTGC---TAGTTCTTTTTCTAAGGGAAAAACGGGA
ACACIA_LIGULATA                  CACGCTAGGGTAATGATACATACACCTATAGGTGATATTTGTCCTAAGACACTATCGGGA
NC_023090_LUPINUS_LUTEUS         CACGCTAGGGTAATGATCCATCAACCCGC---CAGTTCCTTTTATGAGGCACAAACGGGA
AP002983_LOTUS_JAPONICUS         CACGCTAGGGTAATGATCCATCAACCTGC---TAGTTCTTTTTATGAGGCACAAACGGGA

                                         430       440       450       460       470       480
                                           |         |         |         |         |         |
KC893635_GLYCINE_CANESCENS       GAATTTATCCTGGAAGCGGAAGAGCTACTGAAAATGCGTGAAACTATCACAAGGGTTTAT
KC893637_GLYCINE_FALCATA         GAATTTATCCTGGAAGCGGAAGAGCTACTGAAAATGCGTGAAACTATCACAAGGGTTTAT
KC893633_GLYCINE_TOMENTELLA      GAATTTATCCTGGAAGCGGAAGAGCTACTGAAAATGCGTGAAACTATCACAAGGGTTTAT
KC893638_GLYCINE_SYNDETIKA       GAATTTATCCTGGAAGCGGAAGAGCTACTGAAAATGCGTGAAACTATCACAAGGGTTTAT
KC893636_GLYCINE_DOLICHOCARPA    GAATTTATCCTGGAAGCGGAAGAGCTACTGAAAATGCGTGAAACTATCACAAGGGTTTAT
KC893634_GLYCINE_STENOPHITA      GAATTTATCCTGGAAGCGGAAGAGCTACTGAAAATGCGTGAAACTATCACAAGGGTTTAT
KC893632_GLYCINE_CYRTOLOBA       GAATTTATCCTGGAAGCGGAAGAGCTACTGAAAATGCGTGAAACTATCACAAGGGTTTAT
KF611800_GLYCINE_SOJA            GAATTTATCCTGGAAGCGGAAGAGCTACTGAAAATGCGTGAAACTATCACAAGGGTTTAT
NC_007942_GLYCINE_MAX            GAATTTATCCTGGAAGCGGAAGAGCTACTGAAAATGCGTGAAACTATCACAAGGGTTTAT
JQ755301_VIGNA_UNGUICULATA       GAATTTGTCCTGGAAGCAGAAGAGCTACTGAAACTGCGTGAAACTATCACAAATGTTTAT
NC_013843_VIGNA_RADIATA          GAATTTGTCCTGGAAGCAGAAGAGCTACTGAAACTGCGTGAAACTATCACAAATGTTTAT
AP012598_VIGNA_ANGULARIS         GAATTTGTCCTGGAAGCAGAAGAGCTACTGAAAATGCGTGAAACTATCACAAGGGTTTAT
NC_009259_PHASEOLUS_VULGARIS     GAATTTATCCTGGAAGCGGAAGAGCTACTGAAAATGCGTGAAACTATCACAAGGGTTTAT
NC_016708_MILLETTIA_PINNATA      GAATTTATCCGGGAAGCGGAAGAACTACTGAAATTGCGTGAAACTATCACAAGGGTTTAT
NC_014057_PISUM_SATIVUM          GAATGTATGCTGGAAGCACACGAATTACTGTCAATTCGACAAACGATGACAAATATTTAT
NC_014063_LATHYRUS_SATIVUS       GAATGTATGCTGGAAGCAGATGAAATGGTGATACTTCGAGAAACAATAACAGCTATTTAT
EU849487_TRIFOLIUM_SUBTERRANEUM  GAATGTATGCTGGAAGCGGATGAATTACTGAAAATGTACATAGGTATAATAAATATTTAT
NC_011163_CICER_ARIETINUM        GAATGTATGCTGGAAGCGAATGAATTACTGCAAATGCGCCGAAATATCACAAATATTTAT
NC_003119_MEDICAGO_TRUNCATULA     GAATGTATGCTGGAAGCGGAGGAATTACTGAAAATGCGAAAAACTATCACAAATATTTAT
```

```
DQ226511_MORUS_INDICA           GAATTTATCCTGGAAGCGGAAGAACTACTGAAGCTGCGCGAAACCATCACAAGGGTTTAT
NC_015996_PYRUS_PYRIFOLIA       GAATTTATCTTGGAAGCGGAAGAACTTTTGAAGCTGCGCGAAACCGTCACAAGGGTTTAT
NC_014674_CASTANEA_MOLLISSIMA   GAATTTATCCTGGAAGCGGAAGAAATGATGAAGCTGCGCGAAACCATTACAGGGGTTTAT
KC180787_EUCALYPTUS_GLOBULUS    GAATTTATCCTGGAAGCAGAAGAACTGCTGAAACTGCGCGAAACCATCACAAGAGTTTAT
DQ119058_CUCUMIS_SATIVUS        GAATTTGTCCTGGAATCGACCGAACTACTGAACCTGCGCGAAACCATCACAAAGGTTTAT
ACACIA_LIGULATA                 GACTATGTCCTGGAATTGGAGGAAGTAGCGAGGTTGTACACGCGTGTCCTAGAGAAATAT
NC_023090_LUPINUS_LUTEUS        GAATTTATCCTGGAAGCGCAAGAACTGTTGAAACTGCGCGAAACGATCACAAGGGTTTAT
AP002983_LOTUS_JAPONICUS        GAATTTATCCTGGAAGCGGAAGAACTCCTGAAACTACGCGAAACTATCACAAGGGTTTAT

                                        490       500       510       520       530       540
                                         |         |         |         |         |         |
KC893635_GLYCINE_CANESCENS      GTACAAAGAACAGGCAAACCTTTATGGGTTATATCCGAAGACATGGAAAGGGATGTTTTT
KC893637_GLYCINE_FALCATA        GTACAAAGAACAGGCAAACCTTTATGGGTTATATCCGAAGACATGGAAAGGGATGTTTTT
KC893633_GLYCINE_TOMENTELLA     GTACAAAGAACAGGCAAACCTTTATGGGTTATATCCGAAGACATGGAAAGGGATGTTTTT
KC893638_GLYCINE_SYNDETIKA      GTACAAAGAACAGGCAAACCTTTATGGGTTATATCCGAAGACATGGAAAGGGATGTTTTT
KC893636_GLYCINE_DOLICHOCARPA   GTACAAAGAACAGGCAAACCTTTATGGGTTATATCCGAAGACATGGAAAGGGATGTTTTT
KC893634_GLYCINE_STENOPHITA     GTACAAAGAACAGGCAAACCTTTATGGGTTATATCCGAAGACATGGAAAGGGATGTTTTT
KC893632_GLYCINE_CYRTOLOBA      GTACAAAGAACAGGCAAACCTTTATGGGTTATATCCGAAGACATGGAAAGGGATGTTTTT
KF611800_GLYCINE_SOJA           GTACAAAGAACGGGCAAACCTTTATGGGTTATATCCGAAGACATGGAAAGGGATGTTTTT
NC_007942_GLYCINE_MAX           GTACAAAGAACGGGCAAACCTTTATGGGTTATATCCGAAGACATGGAAAGGGATGTTTTT
JQ755301_VIGNA_UNGUICULATA      GTACAAAGAACGGGCAAACCTTTATGGGTTATATCGGACGACATGGAAAGGGACGTTTTT
NC_013843_VIGNA_RADIATA         GTACAAAGAACGGGCAAACCTTTATGGGTTATATCGGACGACATGGAAAGGGACGTTTTT
AP012598_VIGNA_ANGULARIS        GTACAAAGAACGGGCAAACCTTTATGGGTTATATCGGACGACATGGAAAGGGATGTTTTT
NC_009259_PHASEOLUS_VULGARIS    GTACAAAGAACGGGCAAATCTTTATGGGTTATATCGGAAGACATGGAAAGGGATGTTTTT
NC_016708_MILLETTIA_PINNATA     GCACAAAGAACGGGCAAACCCTTATGGGTTGTATCCGAAGATATGGAAAGGGATGTTTTT
NC_014057_PISUM_SATIVUM         TGCCAAAGAACACGCAAACCTTCCTGGCAGATATCTAAAGACCTGGACAGGGATTTTTTT
NC_014063_LATHYRUS_SATIVUS      TCGCAAAGAACACGCAAACCGGTATGGCAGATATCTAAAGACCTGGAAAGGGATCATTTT
EU849487_TRIFOLIUM_SUBTERRANEUM GTACAAAGAACAGGTAAACCCGTATGGCAAATAGCCGTAGACATCAAAAGGGATATTTTT
NC_011163_CICER_ARIETINUM       GCACAAAGAACAGGCAAACCTTCATGGCAGATATCCAAAGACATGGAAAGGGATCTTTTT
NC_003119_MEDICAGO_TRUNCATULA   GCACAAAGAACAGGCAAAGCTTCCTGGCAGATATACAGAGACATGGAGCGGGATCTTTTT
DQ226511_MORUS_INDICA           GTACAAAGAACGGGCAAATCCTTATGGGTTATTTCCGAAGACATGGAAAGAGATGTTTTT
NC_015996_PYRUS_PYRIFOLIA       GTACAAAGGACAGGCAAACCCTTATGGGTTGTATCCGAAGATATGGAAAGAGATGTTTTT
NC_014674_CASTANEA_MOLLISSIMA   GTACAAAGAACAGGCACACCTCTATGGGTTGTATCCGAAGACATGGAAAGAGATGTTTTT
KC180787_EUCALYPTUS_GLOBULUS    GTACAAAGAACGGGCAAACCCCTATGGGTTGTATCCGAAGATATGGAAAGGGATGTTTTT
DQ119058_CUCUMIS_SATIVUS        GTCCAAAGAACAGGCAAACCCTTATGGGTTATATCCGAAGACCTGGAAAGAGATGTTTTT
ACACIA_LIGULATA                 ACAATCAGAACGGGCAAAACTCTACCTGTTATACTGAAAGACATGGAAAAAGTTGTCTTT
NC_023090_LUPINUS_LUTEUS        GTACAAAGAACGGGCAAACCTTTATGGCTTGTATCTGAAGACATGGAAAGGGATGTTTTT
AP002983_LOTUS_JAPONICUS        GTACAAAGAACGGGCAAACCTTTATGGCTTGTATCAGAAGACATGGAAAGGGATGTTTTT

                                        550       560       570       580       590       600
                                         |         |         |         |         |         |
KC893635_GLYCINE_CANESCENS      ATGTCAGCAGCAGAAGCCCAAGCTCACGGAATTGTAGATCTTGTAGCCGTTGAA------
KC893637_GLYCINE_FALCATA        ATGTCAGCAGCAGAAGCCCAAGCTCACGGAATTGTAGATCTTGTAGCCGTTGAA------
KC893633_GLYCINE_TOMENTELLA     ATGTCAGCAGCAGAAGCCCAAGCTCACGGAATTGTAGATCTTGTAGCCGTTGAA------
KC893638_GLYCINE_SYNDETIKA      ATGTCAGCAGCAGAAGCCCAAGCTCACGGAATTGTAGATCTTGTAGCCGTTGAA------
KC893636_GLYCINE_DOLICHOCARPA   ATGTCAGCAGCAGAAGCCCAAGCTCACGGAATTGTAGATCTTGTAGCCGTTGAA------
KC893634_GLYCINE_STENOPHITA     ATGTCAGCAGCAGAAGCCCAAGCTCACGGAATTGTAGATCTTGTAGCCGTTGAA------
KC893632_GLYCINE_CYRTOLOBA      ATGTCAGCAGCAGAAGCCCAAGCTCACGGAATTGTAGATCTTGTAGCCGTTGAA------
KF611800_GLYCINE_SOJA           ATGTCAGCAGCAGAAGCCCAAGCTCACGGAATTGTAGATCTTGTAGCCGTTGAA------
NC_007942_GLYCINE_MAX           ATGTCAGCAGCAGAAGCCCAAGCTCACGGAATTGTAGATCTTGTAGCCGTTGAA------
JQ755301_VIGNA_UNGUICULATA      ATGTCAGCAGCAGAAGCCCAAGCTCATGGAATTGTAGATCTTGTAGCAGTT---------
NC_013843_VIGNA_RADIATA         ATGTCAGCAGCAGAAGCCCAAGCTCATGGAATTGTAGATCTTGTAGCAGTT---------
AP012598_VIGNA_ANGULARIS        ATGTCAGCAGCAGAAGCCCAAGCTCATGGAATTGTAGATCTTGTAGCAGTT---------
NC_009259_PHASEOLUS_VULGARIS    ATGTCAGCAGCAGAAGCCCAAGCTCATGGAATTGTAGATCTTGTAGCAGTTGAA------
NC_016708_MILLETTIA_PINNATA     ATGTCAGCAGTAGAAGCCCAAGCCCACGGAATTATTGATATTGTAGCAGTTGAA------
NC_014057_PISUM_SATIVUM         ATGTCAGCAGAAGAAGCCCAAGCCTACGGAATTATTGATACAATAGCAGATTCTTGTTCT
NC_014063_LATHYRUS_SATIVUS      ATGTCACCCGAAGAAGCCCAAGCCTACGGAATTATTGATTCCGTATCAGATTCTTTGCCT
EU849487_TRIFOLIUM_SUBTERRANEUM ATGTCAGCAGAAGAAGCTCAAGCCTACGGAATTGTTGATATGGTAGCAGATGCCGATTTT
NC_011163_CICER_ARIETINUM       ATGTCAGCAGAAGAAGCTCAAGCCTACGGACTTGTTGATATGGTAGCGGATTCTTTG---
NC_003119_MEDICAGO_TRUNCATULA   ATGTCAGCAGAAGAAGCTGAAGCCCACGGAATTGTTGATACGGTCGCAGCT---------
DQ226511_MORUS_INDICA           ATGTCAGCAACAGAAGCCCAAGCTCACGGACTTGTTGATCTTGTAGCGGTTGAA------
NC_015996_PYRUS_PYRIFOLIA       ATGTCAGCAACAGAAGCCCAAGCTCATGGAATTGTTGATCTTGTAGCGACTGAA------
NC_014674_CASTANEA_MOLLISSIMA   ATGTCAGCAACAGAAGCCCAAGCTCATGGAATTGTTGATCATGTAGGGGTAGAA------
KC180787_EUCALYPTUS_GLOBULUS    ATGTCAGCAACAGAAGCCCAAGCTCATGGAATTGTTGATCTTGTAGCGATTGAA------
DQ119058_CUCUMIS_SATIVUS        ATGTCAGCACCAGAAGCCCAAGCTCATGGAATTGTTGATCTTGTAGCGGTT---------
ACACIA_LIGULATA                 ATGACAGCAGAAGAAGCCCAAGATCATGGAATTATTGATGATATACTTTTGAACGAAGAT
NC_023090_LUPINUS_LUTEUS        ATGTCCGCAGCAGAAGCCCAAGCCCACGGAATTGTGGATCTTGTAGCGGTTGAA------
AP002983_LOTUS_JAPONICUS        ATGTCAGCAGCAGAAGCCCAAGCCTATGGAATTGTTGATCTTGTAGCGGTTGAA------

                                        610       620       630       640
                                         |         |         |         |

KC893635_GLYCINE_CANESCENS      ---------------------------------------TAA
KC893637_GLYCINE_FALCATA        ---------------------------------------TAA
KC893633_GLYCINE_TOMENTELLA     ---------------------------------------TAA
KC893638_GLYCINE_SYNDETIKA      ---------------------------------------TAA
KC893636_GLYCINE_DOLICHOCARPA   ---------------------------------------TAA
```

```
KC893634_GLYCINE_STENOPHITA     ---------------------------------------TAA
KC893632_GLYCINE_CYRTOLOBA      ---------------------------------------TAA
KF611800_GLYCINE_SOJA           ---------------------------------------TAA
NC_007942_GLYCINE_MAX           ---------------------------------------TAA
JQ755301_VIGNA_UNGUICULATA      ---------------------------------------TAA
NC_013843_VIGNA_RADIATA         ---------------------------------------TAA
AP012598_VIGNA_ANGULARIS        ---------------------------------------TAA
NC_009259_PHASEOLUS_VULGARIS    ---------------------------------------TAA
NC_016708_MILLETTIA_PINNATA     ---------------------------------------TAA
NC_014057_PISUM_SATIVUM         TGG------------------------------------TAA
NC_014063_LATHYRUS_SATIVUS      ACGGAAGCAATTTTTGATTCCGGAGCAGATTCTTTTTTTTAA
EU849487_TRIFOLIUM_SUBTERRANEUM TTG------------------------------------TAA
NC_011163_CICER_ARIETINUM       ---------------------------------------TGA
NC_003119_MEDICAGO_TRUNCATULA   ---------------------------------------TAA
DQ226511_MORUS_INDICA           ---------------------------------------TAA
NC_015996_PYRUS_PYRIFOLIA       ---------------------------------------TAA
NC_014674_CASTANEA_MOLLISSIMA   ---------------------------------------TAA
KC180787_EUCALYPTUS_GLOBULUS    ---------------------------------------TAA
DQ119058_CUCUMIS_SATIVUS        ---------------------------------------TAA
ACACIA_LIGULATA                 AAATATTGGGAATGGGAAGAAGATGTA------------TAG
NC_023090_LUPINUS_LUTEUS        ---------------------------------------TAA
AP002983_LOTUS_JAPONICUS        ---------------------------------------TAA
```
