# Supplementary material for: The Complete Sequence of the Acacia ligulata Chloroplast Genome Reveals a Highly Divergent clpP1 Gene
Source: PLoS One. 2015 May 8;10(5):e0125768. doi: 10.1371/journal.pone.0125768 (PMC4425659; doi:10.1371/journal.pone.0125768)
Supplement: S1 Table — The table lists repeated sequences of 30 or more nucleotides in length. The type of repeat (C, complementary; P, palindromic; F, forward; R, reverse) is indicated. (DOCX) [file pone.0125768.s003.docx]

Table S1. Repeated Sequences in the Chloroplast Genome of *Acacia ligulata*

| **First repeat copy** | | **Second repeat copy** | | **Mis-match** | **Type** | **Size** | **Distance between repeats** |
| --- | --- | --- | --- | --- | --- | --- | --- |
| **Start** | **Location** | **Start** | **Location** |  |  |  |  |
| 1728 | *psbA-trnK* | 82065 | *petD-rpoA* | -3 | C | 30 | 80,337 |
| 4823 | *trnK-trnQ* | 4833 | *trnK-trnQ* | -3 | P | 31 | 10 |
| 4910 | *trnK-trnQ* | 82072 | *petD-rpoA* | -3 | P | 30 | 77,162 |
| 4914 | *trnK-trnQ* | 4915 | *trnK-trnQ* | -1 | F | 30 | 1 |
| 4914 | *trnK-trnQ* | 4927 | *trnK-trnQ* | -2 | R | 31 | 13 |
| 4914 | *trnK-trnQ* | 4916 | *trnK-trnQ* | -3 | F | 31 | 2 |
| 4914 | *trnK-trnQ* | 4926 | *trnK-trnQ* | -3 | R | 31 | 12 |
| 4914 | *trnK-trnQ* | 82078 | *petD-rpoA* | -3 | F | 31 | 77,164 |
| 4915 | *trnK-trnQ* | 4928 | *trnK-trnQ* | -2 | R | 30 | 13 |
| 4931 | *trnK-trnQ* | 82072 | *petD-rpoA* | -3 | C | 31 | 77,141 |
| 5221 | *trnK-trnQ* | 5260 | *trnK-trnQ* | -3 | F | 30 | 39 |
| 5247 | *trnK-trnQ* | 88281 | *rps3-rps19* | -3 | F | 30 | 83,034 |
| 5292 | *trnK-trnQ* | 5292 | *trnK-trnQ* | 0 | P | 30 | - |
| 5554 | *trnK-trnQ* | 54067 | *trnK-trnQ* | -3 | F | 30 | 48,513 |
| 7891 | *trnQ-psbK* | 54088 | *aptB* | -3 | F | 31 | 46,197 |
| 13632 | *atpF-atpH* | 69357 | *psbE-petL* | -3 | P | 30 | 55,725 |
| 17497 | *rps2-rpoC2* | 82070 | *petD-rpoA* | -3 | F | 31 | 64,573 |
| 29874 | *trnC-petN* | 62781 | *accD-psaI* | -3 | P | 31 | 32,907 |
| 29977 | *trnC-petN* | 30155 | *trnC-petN* | -1 | F | 48 | 178 |
| 31558 | *petN-psbM* | 31558 | *petN-psbM* | -3 | P | 33 | - |
| 39052 | *psbZ-trnG* | 74800 | *clpP* intron | -3 | P | 30 | 35,748 |
| 39057 | *psbZ-trnG* | 74792 | *clpP* intron | -3 | C | 31 | 35,735 |
| 39138 | *psbZ-trnG* | 39150 | *psbZ-trnG* | -3 | F | 33 | 12 |
| 41567 | *psaB* | 43791 | *psaA* | -3 | F | 41 | 2,224 |
| 45465 | *psaA-ycf3* | 45468 | *psaA-ycf3* | -1 | R | 30 | 3 |
| 46682 | *ycf3* intron | 86012 | *rpl16* intron | -2 | F | 39 | 39,330 |
| 46682 | *ycf3* intron | 103223 | *rps12-trnV* | -2 | F | 39 | 56,541 |
| 46682 | *ycf3* intron | 122874 | *ndhA* intron | -2 | P | 39 | 76,192 |
| 46694 | *ycf3* intron | 103235 | *rps12-trnV* | -3 | F | 30 | 56,541 |
| 50025 | *trnT-trnL* | 50053 | *trnT-trnL* | -1 | F | 30 | 28 |
| 54226 | *ndhC-trnV* | 54259 | *ndhC-trnV* | -3 | P | 30 | 33 |
| 55797 | *trnM-atpE* | 55821 | *trnM-atpE* | -3 | P | 32 | 24 |
| 60617 | *rbcL-accD* | 60617 | *rbcL-accD* | -2 | P | 38 | - |
| 61860 | *accD* | 61920 | *accD* | -3 | F | 34 | 60 |
| 61868 | *accD* | 61904 | *accD* | -3 | F | 49 | 36 |
| 61868 | *accD* | 61916 | *accD* | -3 | F | 37 | 48 |
| 61873 | *accD* | 61897 | *accD* | -2 | F | 56 | 24 |
| 61883 | *accD* | 61895 | *accD* | -2 | F | 58 | 12 |
| 61895 | *accD* | 61907 | *accD* | 0 | F | 46 | 12 |
| 61895 | *accD* | 61919 | *accD* | 0 | F | 34 | 24 |
| 67173 | *petA-psbJ* | 67233 | *petA-psbJ* | 0 | F | 60 | 60 |
| 70867 | *trnP-psaJ* | 82070 | *petD-rpoA* | -3 | P | 31 | 11,203 |
| 72014 | *rpl33-rps18* | 72014 | *rl33-rps18* | -3 | P | 51 | - |
| 72048 | *rpl33-rps18* | 129901 | *rpl32-ndhF* | -3 | R | 31 | 57,853 |
| 74708 | *clpP* intron | 74729 | *clpP* intron | 0 | F | 63 | 21 |
| 74708 | *clpP* intron | 74750 | *clpP* intron | 0 | F | 42 | 42 |
| 74787 | *clpP* intron | 74789 | *clpP* intron | -1 | R | 30 | 2 |
| 74787 | *clpP* intron | 74798 | *clpP* intron | -2 | F | 30 | 11 |
| 74787 | *clpP* intron | 74799 | *clpP* intron | -3 | C | 31 | 12 |
| 78404 | *psbT-psbN* | 78404 | *psnT-psbN* | 0 | P | 44 | - |
| 86000 | *rpl16* intron | 122873 | *ndhA* intron | -2 | P | 52 | 36,873 |
| 86009 | *rpl16* intron | 103220 | *rps12-trnV* | 0 | F | 43 | 17,211 |
| 88293 | *rps3-rps19* | 88293 | *rps3-rps19* | -2 | P | 32 | - |
| 90618 | *rpl23-trnI* | 90689 | *rpl23-trnI* | -2 | F | 54 | 71 |
| 103221 | *rps12-trnV* | 122873 | *ndhA* intron | 0 | P | 42 | 19,652 |
| 113931 | *trnN-ycf1* | 113967 | *trnN-ycf1* | -3 | F | 30 | 36 |
| 114655 | *ycf1* | 114655 | *ycf1* | -2 | P | 32 | - |
| 127818 | *ndhD-ccsA* | 127818 | *ndhD-ccsA* | -2 | P | 42 | - |
| 129338 | *trnL-rpl32* | 129338 | *trnL-rpl32* | -2 | R | 31 | - |
