# Supplementary material for: The Complete Sequence of the Acacia ligulata Chloroplast Genome Reveals a Highly Divergent clpP1 Gene
Source: PLoS One. 2015 May 8;10(5):e0125768. doi: 10.1371/journal.pone.0125768 (PMC4425659; doi:10.1371/journal.pone.0125768)
Supplement: S2 Table — (DOCX) [file pone.0125768.s004.docx]

**Table S2. Tandem repeat sequences in the *Acacia ligulata* chloroplast genome.**

|  | **Size (bp)** | **Repeats** | **Start** | **End** | **Sequence** | **Location** |
| --- | --- | --- | --- | --- | --- | --- |
| 1 | 10 | 2 | 60,069 | 60,090 | TTGTATATAT | *rbcL-accD* |
| 2 | 10 | 2 | 104,046 | 104,067 | ATATGATCCG | *rps12-trnV* |
| 3 | 10 | 2 | 39,178 | 39,198 | ATAGATATAG | *psbZ-trnG* |
| 4 | 10 | 2 | 29,548 | 29,567 | ATTCTATAAA | *trnC-petN* |
| 5 | 10 | 2 | 38,924 | 38,943 | AAATATATTC | *psbZ-trnG* |
| 6 | 10 | 2 | 60,052 | 60,071 | GATACTATTG | *rbcL-accD* |
| 7 | 11 | 3 | 74,788 | 74,817 | TATATTATATA | *clpP1* intron |
| 8 | 11 | 3 | 45,468 | 45,495 | TAATAATAAAA | *psaA-ycf3* |
| 9 | 11 | 2 | 60,612 | 60,634 | TTTCAATTCTA | *rbcL-accD* |
| 10 | 11 | 2 | 13,327 | 13,348 | TAATTTATTCA | *atpF* intron |
| 11 | 11 | 2 | 28,850 | 28,871 | AATCTATTTAA | *rpoB-trnC* |
| 12 | 11 | 2 | 52,595 | 52,616 | TTTGTATAAGT | *ndhJ-ndhK* |
| 13 | 11 | 2 | 104,140 | 104,161 | ATTAGTATTAG | *rps12-trnV^A^* |
| 14 | 11 | 2 | 121,855 | 121,876 | TTTATAGGAAA | *ndhA* intron |
| 15 | 11 | 2 | 124,108 | 124,129 | ATAGTTTCGAA | *ndhI-ndhG* |
| 16 | 12 | 5 | 61,896 | 61,953 | GGAATCTTATGA | *accD* |
| 17 | 12 | 3 | 39,148 | 39,183 | TATTTTATAGAT | *psbZ-trnG* |
| 18 | 12 | 2 | 88,292 | 88,319 | AATATATATATT | *rps3-rps19* |
| 19 | 12 | 2 | 7,135 | 7,158 | AGAAATATATAA | *trnK-trnQ* |
| 20 | 12 | 2 | 81,206 | 81,229 | ATAAGAACATAA | *petD* intron |
| 21 | 12 | 2 | 90,968 | 90,991 | ATTGAGAGAGAT | *ycf2^B^* |
| 22 | 13 | 2 | 8,700 | 8,726 | AAATTCTAAATAT | *psbI-trnG* |
| 23 | 13 | 2 | 10,555 | 10,581 | AATTAATAATATA | *trnR-atpA* |
| 24 | 15 | 2 | 72,019 | 72,053 | TATATAACATATAAT | *rpl33-rps18* |
| 25 | 15 | 2 | 95,892 | 95,926 | GATGATGATAGTGAC | *ycf2* |
| 26 | 15 | 2 | 10,505 | 10,535 | ATTTTTAATTAATTA | *trnR-atpA* |
| 27 | 15 | 2 | 90,649 | 90,862 | TTTGGTTTAATTAGT | *rpl23-trnI* |
| 28 | 18 | 2 | 61,828 | 61,863 | GAGAAATCTTATGAAGAA | *accD* |
| 29 | 21 | 4 | 74,709 | 74,792 | ATCAAATGTAAATGCTTATAT | *clpP1* intron |
| 30 | 21 | 2 | 74,066 | 74,107 | GATCATGAATTACTTATATTC | *rps12-clpP* |
| 31 | 24 | 2 | 114,590 | 114,638 | ATTGGTATTAGTCTGGATACAGAA | *ycf1* |
| 32 | 60 | 2 | 67,174 | 67,293 | TTAATAATAATATAATTAATATATTCCTATTACAATTTATTACAATACAATATATTTAAT | *petA-psbJ* |

^A^ Repeat is also found in *Lotus japonicus, Millettia pinnata* and *Lupinus luteus*

^B^ Repeat is also found in *Millettia pinnata* and *Lupinus luteus*
