# Supplementary material for: The Complete Sequence of the Acacia ligulata Chloroplast Genome Reveals a Highly Divergent clpP1 Gene
Source: PLoS One. 2015 May 8;10(5):e0125768. doi: 10.1371/journal.pone.0125768 (PMC4425659; doi:10.1371/journal.pone.0125768)
Supplement: S3 Table — (DOCX) [file pone.0125768.s005.docx]

**Table S3. Primers Used to Fill Gaps in the *Acacia ligulata* Chloroplast Genome Sequence.**

| **Name** | **Direction** | **Primer sequence (5’ to 3’)** | **Annealing temperature** |
| --- | --- | --- | --- |
| Gap_01 | Forward | ACAGATCGTATGGTAGGACA | 64ºC |
|  | Reverse | GCGTATTTGCGTCTTTGATA |  |
| Gap_02 | Forward | TGCCTTCGCCATATCAATAT | 51ºC |
|  | Reverse | ACGGCTCTACTATGGAATTG |  |
| Gap_03 | Forward | TCGTACGAGATAGAGGAACC | 50ºC |
|  | Reverse | GGGGATTTTGTGACATTTCG |  |
| Gap_04 | Forward | TCAAAACATCTCTTCCTCGA | 65ºC |
|  | Reverse | GTAGAGTAGTCGACAAACAA |  |
| Gap_05 | Forward | GGTACCTCGATTTAATATTTGT | 65ºC |
|  | Reverse | GGGCAAAAGAGTAATTGAGC |  |
| Gap_06 | Forward | GGATTAATTGTGCATCCAAC | 65ºC |
|  | Reverse | TCCAGTAATTACCGTTCGTT |  |
| Gap_07 | Forward | CTACGTCAGGATAACTCTTC | 65ºC |
|  | Reverse | AGATCTACTCCTATGAATGTGG |  |
| Gap_08 | Forward | GCGTCTTCTCTTTGGCAAA | 51ºC |
|  | Reverse | GGGGTCAAACTTCTGGAAA |  |
| Gap_09 | Forward | CACCTTTGGAAGTATTAAGGG | 51ºC |
|  | Reverse | AAAGTATATGAGCACTCCGG |  |
| Gap_10 | Forward | AAAGATTACCGGGGAATTGT | 51ºC |
|  | Reverse | TTTTCACGAGCCCATATG |  |
| Gap_11 | Forward | GCATACTAACTCGCCTTCTT | 66ºC |
|  | Reverse | TATTAAACCCGAAACTCCCG |  |
| Gap_12 | Forward | TCTTGTTGAGTTACGTGCTT | 51ºC |
|  | Reverse | GAGGTCTTCTAAACCTTTGG |  |
| Gap_13 | Forward | GGAATTAAGAAAAGAGGACCC | 53ºC |
|  | Reverse | CGGATTCCTATCTAACGATCC |  |
| Gap_14 | Forward | TTCAGGTAATTTCGCGAAGA | 50ºC |
|  | Reverse | TTTGTCAATCCCAGTCCAAA |  |
| Gap_15 | Forward | GGTTTTGGTCCCGCTATT | 51ºC |
|  | Reverse | ATCATGTCTTTCAAGTCGCA |  |
| Gap_16 | Forward | AAGAGAGGGGAGAGATCTTC | 65ºC |
|  | Reverse | TTTCTCAGATAACACTCAGA |  |
| Gap_17 | Forward | CGATTTCCTTCCCTATCAG | 49ºC |
|  | Reverse | CCCAACTCATAATTGGCGA |  |
| Gap_18 | Forward | TCCGTTCCATGCCTCATT | 51ºC |
|  | Reverse | GGGGTAGCCGAATTTCTTC |  |
| Gap_19 | Forward | ACGCCTTTTGAATTATGGATC | 65ºC |
|  | Reverse | TGGTCATATAATCGTGGTTACA |  |
| Gap_20 | Forward | TTGGGCGTTTATTACTTGGA | 50ºC |
|  | Reverse | CTCATTATCAGTTGACAAGGTC |  |
| Gap_21 | Forward | TGGTGTTTCTAACCATCCAC | 51ºC |
|  | Reverse | GGAATTCGGATTGATGAACT |  |
