# Supplementary material for: The Complete Sequence of the Acacia ligulata Chloroplast Genome Reveals a Highly Divergent clpP1 Gene
Source: PLoS One. 2015 May 8;10(5):e0125768. doi: 10.1371/journal.pone.0125768 (PMC4425659; doi:10.1371/journal.pone.0125768)
Supplement: S4 Table — (DOCX) [file pone.0125768.s006.docx]

**Table S4. Models, Gamma Distribution and Proportion of Invariant Sites, as Estimated by jModelTest for Each Gene Alignment.**

| **Gene** | **Model** | **Gamma** | **Invariant sites** |
| --- | --- | --- | --- |
| *accD* | GTR | 0.39787 |  |
| *atpA* | GTR | 0.20121 | 0.1 |
| *atpB* | GTR | 0.19666 |  |
| *atpE* | GTR | 0.39295 |  |
| *atpF* | GTR | 0.39295 |  |
| *atpH* | GTR | 0.10486 |  |
| *atpI* | GTR | 0.24053 |  |
| *ccsA* | GTR | 0.56555 |  |
| *cemA* | GTR | 0.93733 |  |
| *clpP1* | GTR | 0.88189 |  |
| *matK* | GTR | 1.10201 |  |
| *ndhA* | GTR | 0.31989 |  |
| *ndhB* | GTR | 0.1288 |  |
| *ndhC* | GTR | 0.20386 |  |
| *ndhD* | GTR | 0.37045 |  |
| *ndhE* | GTR | 0.1866 |  |
| *ndhF* | GTR | 0.47714 |  |
| *ndhG* | GTR | 0.43538 |  |
| *ndhH* | GTR | 0.16256 |  |
| *ndhI* | HKY85 | 0.34485 |  |
| *ndhJ* | GTR | 0.22452 |  |
| *ndhK* | GTR | 0.30079 |  |
| *petA* | GTR | 0.26695 |  |
| *petB* | GTR | 0.05641 |  |
| *petD* | GTR | 0.06651 | 0.1 |
| *petG* | GTR | 0.36945 |  |
| *petL* | HKY85 | 0.60661 |  |
| *petN* | HKY85 | 0.34975 |  |
| *psaA* | GTR | 0.17483 |  |
| *psaB* | GTR | 0.11306 |  |
| *psaC* | HKY85 | 0.05658 |  |
| *psaI* | HKY85 | 0.61993 |  |
| *psaJ* | HKY85 | 0.20277 |  |
| *psbA* | GTR | 0.09718 |  |
| *psbB* | GTR | 0.09585 |  |
| *psbC* | GTR | 0.05695 | 0.1 |
| *psbD* | GTR | 0.11326 | 0.1 |
| *psbE* | TN93 |  |  |
| *psbF* | GTR | 0.24800 |  |
| *psbH* | HKY85 | 0.11326 |  |
| *psbI* | HKY85 | 0.17295 |  |
| *psbJ* | HKY85 | 0.10379 |  |
| *psbK* | GTR | 0.27066 |  |
| *psbL* | HKY85 | 0.05586 |  |
| *psbM* | HKY85 | 0.52773 |  |
| *psbN* | K2P | 0.18359 |  |
| *psbT* | HKY85 | 0.21824 |  |
| *psbZ* | GTR | 0.2991 | 0.1 |
| *rbcL* | TN93 | 0.11267 | 0.1 |
| *rpl2* | GTR | 0.28493 |  |
| *rpl14* | GTR | 0.37351 |  |
| *rpl16* | GTR | 0.31267 |  |
| *rpl20* | GTR | 0.48327 |  |
| *rpl23* | GTR | 0.24468 |  |
| *rpl32* | GTR | 0.38636 |  |
| *rpl33* | GTR | 0.81216 |  |
| *rpl36* | GTR | 0.22947 |  |
| *rpoA* | GTR | 0.53863 |  |
| *rpoB* | GTR | 0.33804 |  |
| *rpoC1* | GTR | 0.37697 |  |
| *rpoC2* | GTR | 0.55584 |  |
| *rps2* | GTR | 0.49741 |  |
| *rps3* | GTR | 0.83524 |  |
| *rps4* | GTR | 0.43778 |  |
| *rps7* | GTR | 0.38112 |  |
| *rps8* | GTR | 0.62478 |  |
| *rps11* | GTR | 0.4081 |  |
| *rps12* | HKY85 | 0.15654 |  |
| *rps14* | GTR | 0.46228 |  |
| *rps15* | GTR | 0.99738 |  |
| *rps18* | GTR | 0.22876 |  |
| *rps19* | GTR | 0.38645 |  |
| *ycf1* | GTR | 0.84114 | 0.1 |
| *ycf2* | GTR | 0.37192 |  |
| *ycf3* | GTR | 0.19976 |  |
| *ycf4* | GTR | 0.53501 |  |
| *Concatenation of all genes* | GTR | 0.37957 | 0.1 |

GTR = General Time Reversible; HKY85 = Hasegawa, Kishino and Yano (1985); TN93 = Timura and Nei (1993); K2P = Kimura 2-parameter
