# Supplementary material for: The Complete Sequence of the Acacia ligulata Chloroplast Genome Reveals a Highly Divergent clpP1 Gene
Source: PLoS One. 2015 May 8;10(5):e0125768. doi: 10.1371/journal.pone.0125768 (PMC4425659; doi:10.1371/journal.pone.0125768)
Supplement: S5 Table — (DOCX) [file pone.0125768.s007.docx]

**Table S5. Primers Used to Test for *clpP* Intron Splicing in *Acacia ligulata.***

| **Name** | **Location** | **Direction** | **Sequence (5’ to 3’)** |
| --- | --- | --- | --- |
| Primer_A | Exon 1 | Forward | GAAGATGAAAACGCGTCTTGG |
| Primer_C | Exon 2 | Reverse | TACCATCCATCCACCTGGAC |
| Primer_D | Exon 2 | Forward | AATATGCGTGGGAACAGC |
| Primer_F | Exon 3 | Reverse | GCTACTTCCTCCAATTCC |
