# Supplementary material for: Correction: The Complete Sequence of the Acacia ligulata Chloroplast Genome Reveals a Highly Divergent clpP1 Gene
Source: PLoS One. 2015 Sep 14;10(9):e0138367. doi: 10.1371/journal.pone.0138367 (PMC4569417; doi:10.1371/journal.pone.0138367)
Supplement: S1 Table — The table lists repeated sequences of 30 or more nucleotides in length. The type of repeat (C, complementary; P, palindromic; F, forward; R, reverse) is indicated. (DOCX) [file pone.0138367.s001.docx]

S1 Table. Repeated Sequences in the Chloroplast Genome of *Acacia ligulata*

| **First repeat copy** | | **Second repeat copy** | | **Mis-match** | **Type** | **Size** | **Distance between repeats** |
| --- | --- | --- | --- | --- | --- | --- | --- |
| **Start** | **Location** | **Start** | **Location** |  |  |  |  |
| 0 | *trnI-trnH* | 90724 | *rps19* gene | 0 | P | 49 | 90724 |
| 1784 | *psbA-trnK* | 84165 | *petD-rpoA* | -3 | C | 30 | 82381 |
| 4915 | *trnK-trnQ* | 4915 | *trnK-trnQ* | -2 | R | 31 | 0 |
| 4984 | *trnK-trnQ* | 4996 | *trnK-trnQ* | -3 | R | 33 | 12 |
| 5002 | *trnK-trnQ* | 5006 | *trnK-trnQ* | -3 | F | 30 | 4 |
| 5003 | *trnK-trnQ* | 5013 | *trnK-trnQ* | -3 | R | 33 | 10 |
| 5015 | *trnK-trnQ* | 11086 | *trnG-trnR* | -3 | F | 30 | 6071 |
| 5313 | *trnK-trnQ* | 5352 | *trnK-trnQ* | -3 | F | 30 | 39 |
| 5339 | *trnK-trnQ* | 90433 | *rps3-rps19* | -3 | F | 30 | 85094 |
| 5384 | *trnK-trnQ* | 5384 | *trnK-trnQ* | 0 | P | 30 | 0 |
| 5650 | *trnK-trnQ* | 55468 | *ndhC-trnV* | -3 | F | 30 | 49818 |
| 5668 | *trnK-trnQ* | 61581 | *rbcL-accD* | -3 | F | 30 | 55913 |
| 7996 | *trnQ-psbK* | 55489 | *ndhC-trnV* | -3 | F | 31 | 47493 |
| 8948 | *psbI-trnS* | 8948 | *psbI-trnS* | -1 | P | 37 | 0 |
| 9079 | *psbI-trnS* | 39300 | *psbC-trnS* | -3 | F | 32 | 30221 |
| 9081 | *psbI-trnS* | 49050 | *trnS-GGA* | -1 | P | 30 | 39969 |
| 9269 | *trnS-trnG* | 9269 | *trnS-trnG* | 0 | P | 32 | 0 |
| 11699 | *trnR-atpA* | 31130 | *trnC-petN* | -3 | P | 34 | 19431 |
| 14730 | *atpF-atpA* | 71319 | *psbE-petL* | -3 | P | 30 | 56589 |
| 18607 | *rps2-rpoC2* | 84170 | *petD-rpoA* | -3 | F | 31 | 65563 |
| 31012 | *trnC-petN* | 64661 | *accD-psaI* | -3 | P | 31 | 33649 |
| 32763 | *petN-psbM* | 32763 | *petN-psbM* | -3 | P | 33 | 0 |
| 40307 | *psbZ-trnG* | 76855 | *clpP1* intron | -3 | P | 30 | 36548 |
| 40312 | *psbZ-trnG* | 76847 | *clpP1* intron | -3 | C | 31 | 36535 |
| 40393 | *psbZ-trnG* | 40405 | *psbZ-trnG* | -3 | F | 33 | 12 |
| 42823 | *psaB* gene | 45047 | *psaA* gene | -3 | F | 41 | 2224 |
| 47942 | *ycf3* intron | 88122 | *rpl16* intron | -2 | F | 39 | 40180 |
| 47942 | *ycf3* intron | 102695 | *ndhA* intron | -2 | F | 39 | 54753 |
| 47942 | *ycf3* intron | 123292 | *trnV-rps12* | -2 | P | 39 | 75350 |
| 47954 | *ycf3* intron | 123289 | *trnV-rps12* | -3 | P | 30 | 75335 |
| 51384 | *trnT-trnL* | 51412 | *trnT-trnL* | -1 | F | 30 | 28 |
| 55627 | *ndhC-trnV* | 55660 | *ndhC-trnV* | -3 | P | 30 | 33 |
| 57221 | *trnfM-atpE* | 57245 | *trnfM-atpE* | -3 | P | 32 | 24 |
| 61547 | *rbcL-accD* | 61549 | *rbcL-accD* | -1 | R | 31 | 2 |
| 62077 | *rbcL-accD* | 62077 | *rbcL-accD* | -3 | P | 41 | 0 |
| 63287 | *accD* gene | 63359 | *accD* gene | -3 | F | 99 | 72 |
| 63287 | *accD* gene | 63635 | *accD* gene | -3 | F | 75 | 348 |
| 63287 | *accD* gene | 63515 | *accD* gene | -2 | F | 70 | 228 |
| 63287 | *accD* gene | 63575 | *accD* gene | -2 | F | 70 | 288 |
| 63301 | *accD* gene | 63673 | *accD* gene | -2 | F | 61 | 372 |
| 63301 | *accD* gene | 63325 | *accD* gene | -2 | F | 46 | 24 |
| 63301 | *accD* gene | 63397 | *accD* gene | -2 | F | 46 | 96 |
| 63301 | *accD* gene | 63493 | *accD* gene | 0 | F | 32 | 192 |
| 63301 | *accD* gene | 63553 | *accD* gene | 0 | F | 32 | 252 |
| 63301 | *accD* gene | 63613 | *accD* gene | 0 | F | 32 | 312 |
| 63302 | *accD* gene | 63698 | *accD* gene | -1 | F | 51 | 396 |
| 63302 | *accD* gene | 63722 | *accD* gene | -1 | F | 31 | 420 |
| 63315 | *accD* gene | 63759 | *accD* gene | -3 | F | 38 | 444 |
| 63315 | *accD* gene | 63771 | *accD* gene | -3 | F | 38 | 456 |
| 63315 | *accD* gene | 63447 | *accD* gene | -3 | F | 32 | 132 |
| 63315 | *accD* gene | 63783 | *accD* gene | -3 | F | 32 | 468 |
| 63318 | *accD* gene | 63486 | *accD* gene | 0 | F | 39 | 168 |
| 63325 | *accD* gene | 63373 | *accD* gene | -2 | F | 46 | 48 |
| 63325 | *accD* gene | 63529 | *accD* gene | -2 | F | 46 | 204 |
| 63325 | *accD* gene | 63589 | *accD* gene | -2 | F | 46 | 264 |
| 63325 | *accD* gene | 63649 | *accD* gene | -2 | F | 46 | 324 |
| 63326 | *accD* gene | 63698 | *accD* gene | -3 | F | 45 | 372 |
| 63326 | *accD* gene | 63722 | *accD* gene | -1 | F | 31 | 396 |
| 63339 | *accD* gene | 63483 | *accD* gene | -3 | F | 32 | 144 |
| 63339 | *accD* gene | 63759 | *accD* gene | -3 | F | 32 | 420 |
| 63339 | *accD* gene | 63771 | *accD* gene | -3 | F | 32 | 432 |
| 63339 | *accD* gene | 63783 | *accD* gene | -3 | F | 32 | 444 |
| 63342 | *accD* gene | 63450 | *accD* gene | -1 | F | 44 | 108 |
| 63358 | *accD* gene | 63634 | *accD* gene | -3 | F | 85 | 276 |
| 63358 | *accD* gene | 63514 | *accD* gene | 0 | F | 71 | 156 |
| 63358 | *accD* gene | 63574 | *accD* gene | 0 | F | 71 | 216 |
| 63361 | *accD* gene | 63481 | *accD* gene | -2 | F | 44 | 120 |
| 63373 | *accD* gene | 63673 | *accD* gene | -2 | F | 61 | 300 |
| 63373 | *accD* gene | 63397 | *accD* gene | -2 | F | 46 | 24 |
| 63373 | *accD* gene | 63493 | *accD* gene | 0 | F | 32 | 120 |
| 63373 | *accD* gene | 63553 | *accD* gene | 0 | F | 32 | 180 |
| 63373 | *accD* gene | 63613 | *accD* gene | 0 | F | 32 | 240 |
| 63374 | *accD* gene | 63698 | *accD* gene | -1 | F | 51 | 324 |
| 63374 | *accD* gene | 63722 | *accD* gene | -1 | F | 31 | 348 |
| 63387 | *accD* gene | 63759 | *accD* gene | -3 | F | 38 | 372 |
| 63387 | *accD* gene | 63771 | *accD* gene | -3 | F | 38 | 384 |
| 63387 | *accD* gene | 63447 | *accD* gene | -3 | F | 32 | 60 |
| 63387 | *accD* gene | 63783 | *accD* gene | -3 | F | 32 | 396 |
| 63390 | *accD* gene | 63486 | *accD* gene | 0 | F | 39 | 96 |
| 63397 | *accD* gene | 63529 | *accD* gene | -2 | F | 46 | 132 |
| 63397 | *accD* gene | 63589 | *accD* gene | -2 | F | 46 | 192 |
| 63397 | *accD* gene | 63649 | *accD* gene | -2 | F | 46 | 252 |
| 63398 | *accD* gene | 63698 | *accD* gene | -3 | F | 45 | 300 |
| 63398 | *accD* gene | 63722 | *accD* gene | -1 | F | 31 | 324 |
| 63411 | *accD* gene | 63483 | *accD* gene | -3 | F | 32 | 72 |
| 63411 | *accD* gene | 63759 | *accD* gene | -3 | F | 32 | 348 |
| 63411 | *accD* gene | 63771 | *accD* gene | -3 | F | 32 | 360 |
| 63411 | *accD* gene | 63783 | *accD* gene | -3 | F | 32 | 372 |
| 63414 | *accD* gene | 63450 | *accD* gene | -2 | F | 65 | 36 |
| 63426 | *accD* gene | 63510 | *accD* gene | -2 | F | 32 | 84 |
| 63426 | *accD* gene | 63570 | *accD* gene | -2 | F | 32 | 144 |
| 63426 | *accD* gene | 63630 | *accD* gene | -2 | F | 32 | 204 |
| 63430 | *accD* gene | 63742 | *accD* gene | -3 | F | 31 | 312 |
| 63433 | *accD* gene | 63445 | *accD* gene | -3 | F | 32 | 12 |
| 63445 | *accD* gene | 63469 | *accD* gene | -3 | F | 32 | 24 |
| 63447 | *accD* gene | 63543 | *accD* gene | -3 | F | 32 | 96 |
| 63447 | *accD* gene | 63603 | *accD* gene | -3 | F | 32 | 156 |
| 63447 | *accD* gene | 63663 | *accD* gene | -3 | F | 32 | 216 |
| 63462 | *accD* gene | 63510 | *accD* gene | -2 | F | 32 | 48 |
| 63462 | *accD* gene | 63570 | *accD* gene | -2 | F | 32 | 108 |
| 63462 | *accD* gene | 63630 | *accD* gene | -2 | F | 32 | 168 |
| 63466 | *accD* gene | 63742 | *accD* gene | -3 | F | 31 | 276 |
| 63481 | *accD* gene | 63517 | *accD* gene | -2 | F | 44 | 36 |
| 63481 | *accD* gene | 63577 | *accD* gene | -2 | F | 44 | 96 |
| 63481 | *accD* gene | 63637 | *accD* gene | -2 | F | 44 | 156 |
| 63486 | *accD* gene | 63546 | *accD* gene | -1 | F | 159 | 60 |
| 63486 | *accD* gene | 63606 | *accD* gene | -1 | F | 99 | 120 |
| 63486 | *accD* gene | 63714 | *accD* gene | -3 | F | 47 | 228 |
| 63486 | *accD* gene | 63666 | *accD* gene | -1 | F | 39 | 180 |
| 63486 | *accD* gene | 63690 | *accD* gene | -2 | F | 39 | 204 |
| 63486 | *accD* gene | 63762 | *accD* gene | -3 | F | 35 | 276 |
| 63486 | *accD* gene | 63774 | *accD* gene | -3 | F | 35 | 288 |
| 63493 | *accD* gene | 63529 | *accD* gene | 0 | F | 32 | 36 |
| 63493 | *accD* gene | 63589 | *accD* gene | 0 | F | 32 | 96 |
| 63493 | *accD* gene | 63649 | *accD* gene | 0 | F | 32 | 156 |
| 63494 | *accD* gene | 63722 | *accD* gene | -3 | F | 43 | 228 |
| 63494 | *accD* gene | 63698 | *accD* gene | -1 | F | 31 | 204 |
| 63529 | *accD* gene | 63673 | *accD* gene | -2 | F | 56 | 144 |
| 63529 | *accD* gene | 63553 | *accD* gene | 0 | F | 32 | 24 |
| 63529 | *accD* gene | 63613 | *accD* gene | 0 | F | 32 | 84 |
| 63530 | *accD* gene | 63698 | *accD* gene | -3 | F | 63 | 168 |
| 63530 | *accD* gene | 63722 | *accD* gene | -1 | F | 31 | 192 |
| 63543 | *accD* gene | 63759 | *accD* gene | -3 | F | 38 | 216 |
| 63543 | *accD* gene | 63771 | *accD* gene | -3 | F | 38 | 228 |
| 63543 | *accD* gene | 63783 | *accD* gene | -3 | F | 32 | 240 |
| 63553 | *accD* gene | 63589 | *accD* gene | 0 | F | 32 | 36 |
| 63553 | *accD* gene | 63649 | *accD* gene | 0 | F | 32 | 96 |
| 63554 | *accD* gene | 63722 | *accD* gene | -3 | F | 43 | 168 |
| 63554 | *accD* gene | 63698 | *accD* gene | -1 | F | 31 | 144 |
| 63589 | *accD* gene | 63673 | *accD* gene | -2 | F | 56 | 84 |
| 63589 | *accD* gene | 63613 | *accD* gene | 0 | F | 32 | 24 |
| 63590 | *accD* gene | 63698 | *accD* gene | -3 | F | 63 | 108 |
| 63590 | *accD* gene | 63722 | *accD* gene | -1 | F | 31 | 132 |
| 63603 | *accD* gene | 63759 | *accD* gene | -3 | F | 38 | 156 |
| 63603 | *accD* gene | 63771 | *accD* gene | -3 | F | 38 | 168 |
| 63603 | *accD* gene | 63783 | *accD* gene | -3 | F | 32 | 180 |
| 63613 | *accD* gene | 63649 | *accD* gene | 0 | F | 32 | 36 |
| 63614 | *accD* gene | 63722 | *accD* gene | -3 | F | 43 | 108 |
| 63614 | *accD* gene | 63698 | *accD* gene | -1 | F | 31 | 84 |
| 63649 | *accD* gene | 63673 | *accD* gene | -1 | F | 72 | 24 |
| 63650 | *accD* gene | 63698 | *accD* gene | -3 | F | 55 | 48 |
| 63650 | *accD* gene | 63722 | *accD* gene | -1 | F | 31 | 72 |
| 63663 | *accD* gene | 63759 | *accD* gene | -3 | F | 34 | 96 |
| 63663 | *accD* gene | 63771 | *accD* gene | -3 | F | 34 | 108 |
| 63663 | *accD* gene | 63783 | *accD* gene | -3 | F | 32 | 120 |
| 63674 | *accD* gene | 63698 | *accD* gene | 0 | F | 47 | 24 |
| 63674 | *accD* gene | 63722 | *accD* gene | -2 | F | 31 | 48 |
| 63722 | *accD* gene | 63782 | *accD* gene | -3 | F | 34 | 60 |
| 63730 | *accD* gene | 63766 | *accD* gene | -3 | F | 49 | 36 |
| 63730 | *accD* gene | 63778 | *accD* gene | -3 | F | 37 | 48 |
| 63735 | *accD* gene | 63759 | *accD* gene | -2 | F | 56 | 24 |
| 63745 | *accD* gene | 63757 | *accD* gene | -2 | F | 58 | 12 |
| 63757 | *accD* gene | 63769 | *accD* gene | 0 | F | 46 | 12 |
| 63757 | *accD* gene | 63781 | *accD* gene | 0 | F | 34 | 24 |
| 64495 | *accD-psaI* | 64513 | *accD-psaI* | -3 | F | 30 | 18 |
| 69117 | *petA-psbJ* | 69177 | *petA-psbJ* | 0 | F | 60 | 60 |
| 72738 | *trnP-psaJ* | 72770 | *trnP-psaJ* | 0 | F | 32 | 32 |
| 72880 | *trnP-psaJ* | 84170 | *petD-rpoA* | -3 | P | 31 | 11290 |
| 74038 | *rpl33-rps18* | 74038 | *rpl33-rps18* | -3 | P | 51 | 0 |
| 74072 | *rpl33-rps18* | 95668 | *ndhF-rpl32* | -3 | C | 31 | 21596 |
| 76763 | *clpP1* intron | 76784 | *clpP1* intron | 0 | F | 63 | 21 |
| 76763 | *clpP1* intron | 76805 | *clpP1* intron | 0 | F | 42 | 42 |
| 76842 | *clpP1* intron | 76854 | *clpP1* intron | -3 | C | 31 | 12 |
| 76842 | *clpP1* intron | 76853 | *clpP1* intron | -2 | F | 30 | 11 |
| 76842 | *clpP1* intron | 76844 | *clpP1* intron | -1 | R | 30 | 2 |
| 80475 | *psbT-psbN* | 80475 | *psbT-psbN* | 0 | P | 44 | 0 |
| 88110 | *rpl16* intron | 102683 | *ndhA* intron | -2 | F | 52 | 14573 |
| 88119 | *rpl16* intron | 123291 | *trnV-rps12* | 0 | P | 43 | 35172 |
| 90445 | *rps3-rps19* | 90445 | *rps3-rps19* | -2 | P | 32 | 0 |
| 96233 | *rpl32-trnL* | 96233 | *rpl32-trnL* | -2 | R | 31 | 0 |
| 97745 | *ccsA-ndhD* | 97745 | *ccsA-ndhD* | -2 | P | 42 | 0 |
| 102693 | *ndhA* intron | 123291 | *trnV-rps12* | 0 | P | 42 | 20598 |
| 103646 | *ndhA* intron | 103646 | *ndhA* intron | 0 | P | 30 | 0 |
| 111114 | *ycf1* gene | 111138 | *ycf1* gene | -2 | F | 32 | 24 |
| 111293 | *ycf1* gene | 111293 | *ycf1* gene | -2 | P | 32 | 0 |
| 111990 | *ycf1-trnN* | 112404 | *ycf1-trnN* | -2 | F | 67 | 414 |
| 111990 | *ycf1-trnN* | 112445 | *ycf1-trnN* | -2 | F | 67 | 455 |
| 111990 | *ycf1-trnN* | 112486 | *ycf1-trnN* | -2 | F | 67 | 496 |
| 111990 | *ycf1-trnN* | 112527 | *ycf1-trnN* | -2 | F | 67 | 537 |
| 111990 | *ycf1-trnN* | 112568 | *ycf1-trnN* | -2 | F | 44 | 578 |
| 112003 | *ycf1-trnN* | 112376 | *ycf1-trnN* | 0 | F | 54 | 373 |
| 112003 | *ycf1-trnN* | 112081 | *ycf1-trnN* | 0 | F | 31 | 78 |
| 112003 | *ycf1-trnN* | 112216 | *ycf1-trnN* | 0 | F | 31 | 213 |
| 112003 | *ycf1-trnN* | 112264 | *ycf1-trnN* | 0 | F | 31 | 261 |
| 112003 | *ycf1-trnN* | 112168 | *ycf1-trnN* | -1 | F | 31 | 165 |
| 112019 | *ycf1-trnN* | 112055 | *ycf1-trnN* | -2 | F | 33 | 36 |
| 112019 | *ycf1-trnN* | 112141 | *ycf1-trnN* | -2 | F | 33 | 122 |
| 112031 | *ycf1-trnN* | 112202 | *ycf1-trnN* | -3 | F | 32 | 171 |
| 112031 | *ycf1-trnN* | 112250 | *ycf1-trnN* | -3 | F | 32 | 219 |
| 112031 | *ycf1-trnN* | 112362 | *ycf1-trnN* | -3 | F | 32 | 331 |
| 112041 | *ycf1-trnN* | 112330 | *ycf1-trnN* | -2 | F | 30 | 289 |
| 112044 | *ycf1-trnN* | 112130 | *ycf1-trnN* | 0 | F | 45 | 86 |
| 112055 | *ycf1-trnN* | 112392 | *ycf1-trnN* | -2 | F | 33 | 337 |
| 112055 | *ycf1-trnN* | 112433 | *ycf1-trnN* | -2 | F | 33 | 378 |
| 112055 | *ycf1-trnN* | 112474 | *ycf1-trnN* | -2 | F | 33 | 419 |
| 112055 | *ycf1-trnN* | 112515 | *ycf1-trnN* | -2 | F | 33 | 460 |
| 112055 | *ycf1-trnN* | 112556 | *ycf1-trnN* | -2 | F | 33 | 501 |
| 112065 | *ycf1-trnN* | 112315 | *ycf1-trnN* | -2 | F | 30 | 250 |
| 112067 | *ycf1-trnN* | 112202 | *ycf1-trnN* | -1 | F | 70 | 135 |
| 112067 | *ycf1-trnN* | 112250 | *ycf1-trnN* | -1 | F | 46 | 183 |
| 112067 | *ycf1-trnN* | 112362 | *ycf1-trnN* | -1 | F | 45 | 295 |
| 112080 | *ycf1-trnN* | 112167 | *ycf1-trnN* | -1 | F | 57 | 87 |
| 112081 | *ycf1-trnN* | 112581 | *ycf1-trnN* | 0 | F | 43 | 500 |
| 112081 | *ycf1-trnN* | 112417 | *ycf1-trnN* | 0 | F | 31 | 336 |
| 112081 | *ycf1-trnN* | 112458 | *ycf1-trnN* | 0 | F | 31 | 377 |
| 112081 | *ycf1-trnN* | 112499 | *ycf1-trnN* | 0 | F | 31 | 418 |
| 112081 | *ycf1-trnN* | 112540 | *ycf1-trnN* | 0 | F | 31 | 459 |
| 112097 | *ycf1-trnN* | 112344 | *ycf1-trnN* | -1 | F | 40 | 247 |
| 112102 | *ycf1-trnN* | 112189 | *ycf1-trnN* | 0 | F | 35 | 87 |
| 112115 | *ycf1-trnN* | 112153 | *ycf1-trnN* | -3 | F | 30 | 38 |
| 112124 | *ycf1-trnN* | 112327 | *ycf1-trnN* | -2 | F | 32 | 203 |
| 112141 | *ycf1-trnN* | 112392 | *ycf1-trnN* | -2 | F | 33 | 251 |
| 112141 | *ycf1-trnN* | 112433 | *ycf1-trnN* | -2 | F | 33 | 292 |
| 112141 | *ycf1-trnN* | 112474 | *ycf1-trnN* | -2 | F | 33 | 333 |
| 112141 | *ycf1-trnN* | 112515 | *ycf1-trnN* | -2 | F | 33 | 374 |
| 112141 | *ycf1-trnN* | 112556 | *ycf1-trnN* | -2 | F | 33 | 415 |
| 112151 | *ycf1-trnN* | 112315 | *ycf1-trnN* | -2 | F | 32 | 164 |
| 112167 | *ycf1-trnN* | 112215 | *ycf1-trnN* | -1 | F | 81 | 48 |
| 112167 | *ycf1-trnN* | 112263 | *ycf1-trnN* | -1 | F | 33 | 96 |
| 112167 | *ycf1-trnN* | 112375 | *ycf1-trnN* | -1 | F | 32 | 208 |
| 112168 | *ycf1-trnN* | 112581 | *ycf1-trnN* | -1 | F | 43 | 413 |
| 112168 | *ycf1-trnN* | 112417 | *ycf1-trnN* | -1 | F | 31 | 249 |
| 112168 | *ycf1-trnN* | 112458 | *ycf1-trnN* | -1 | F | 31 | 290 |
| 112168 | *ycf1-trnN* | 112499 | *ycf1-trnN* | -1 | F | 31 | 331 |
| 112168 | *ycf1-trnN* | 112540 | *ycf1-trnN* | -1 | F | 31 | 372 |
| 112184 | *ycf1-trnN* | 112344 | *ycf1-trnN* | 0 | F | 63 | 160 |
| 112189 | *ycf1-trnN* | 112237 | *ycf1-trnN* | 0 | F | 59 | 48 |
| 112216 | *ycf1-trnN* | 112581 | *ycf1-trnN* | 0 | F | 43 | 365 |
| 112216 | *ycf1-trnN* | 112417 | *ycf1-trnN* | 0 | F | 31 | 201 |
| 112216 | *ycf1-trnN* | 112458 | *ycf1-trnN* | 0 | F | 31 | 242 |
| 112216 | *ycf1-trnN* | 112499 | *ycf1-trnN* | 0 | F | 31 | 283 |
| 112216 | *ycf1-trnN* | 112540 | *ycf1-trnN* | 0 | F | 31 | 324 |
| 112232 | *ycf1-trnN* | 112344 | *ycf1-trnN* | -1 | F | 63 | 112 |
| 112264 | *ycf1-trnN* | 112581 | *ycf1-trnN* | 0 | F | 32 | 317 |
| 112264 | *ycf1-trnN* | 112417 | *ycf1-trnN* | 0 | F | 31 | 153 |
| 112264 | *ycf1-trnN* | 112458 | *ycf1-trnN* | 0 | F | 31 | 194 |
| 112264 | *ycf1-trnN* | 112499 | *ycf1-trnN* | 0 | F | 31 | 235 |
| 112264 | *ycf1-trnN* | 112540 | *ycf1-trnN* | 0 | F | 31 | 276 |
| 112344 | *ycf1-trnN* | 112597 | *ycf1-trnN* | -2 | F | 30 | 253 |
| 112376 | *ycf1-trnN* | 112417 | *ycf1-trnN* | 0 | F | 195 | 41 |
| 112376 | *ycf1-trnN* | 112458 | *ycf1-trnN* | 0 | F | 154 | 82 |
| 112376 | *ycf1-trnN* | 112499 | *ycf1-trnN* | 0 | F | 113 | 123 |
| 112376 | *ycf1-trnN* | 112540 | *ycf1-trnN* | 0 | F | 72 | 164 |
| 112376 | *ycf1-trnN* | 112581 | *ycf1-trnN* | 0 | F | 31 | 205 |
| 130651 | *ycf2* gene | 130666 | *ycf2* gene | -3 | F | 33 | 15 |
| 135851 | *trnI-trnH* | 135922 | *trnI-trnH* | -2 | F | 54 | 71 |
