# Supplementary material for: Correction: The Complete Sequence of the Acacia ligulata Chloroplast Genome Reveals a Highly Divergent clpP1 Gene
Source: PLoS One. 2015 Sep 14;10(9):e0138367. doi: 10.1371/journal.pone.0138367 (PMC4569417; doi:10.1371/journal.pone.0138367)
Supplement: S2 Table — (DOCX) [file pone.0138367.s002.docx]

**S2 Table. Tandem repeat sequences in the *Acacia ligulata* chloroplast genome.**

|  | **Size (bp)** | **Repeats** | **Start** | **End** | **Sequence** | **Location** |
| --- | --- | --- | --- | --- | --- | --- |
| 1 | 10 | 2 | 30,679 | 30,702 | TAAAATTCTA | *trnC-petN* |
| 2 | 10 | 2 | 34,302 | 34,321 | AATGATATGA | *trnE-trnT* |
| 3 | 10 | 2 | 40,179 | 40,198 | AAATATATTC | *psbZ-trnG* |
| 4 | 10 | 2 | 40,433 | 40,453 | ATAGATATAG | *psbZ-trnG* |
| 5 | 10 | 2 | 50,970 | 50,990 | AATAAATAAT | *trnT-trnL* |
| 6 | 10 | 2 | 51,026 | 51,046 | TCTAAATTAA | *trnT-trnL* |
| 7 | 10 | 2 | 61,482 | 61,501 | GATACTATTG | *rbcL-accD* |
| 8 | 10 | 2 | 61,499 | 61,520 | TTGTATATAT | *rbcL-accD* |
| 9 | 10 | 2 | 61,886 | 61,905 | TTAGTATTTA | *rbcL-accD* |
| 10 | 10 | 2 | 122,488 | 122,509 | ATCGGATCAT | *trnV-rps12* |
| 11 | 11 | 2 | 9,171 | 9,192 | CCAATTGAAAA | *trns-trnG* |
| 12 | 11 | 2 | 14,424 | 14,445 | TAATTTATTCA | *atpF* intron |
| 13 | 11 | 2 | 29,976 | 29,997 | AATCTATTTAA | *rpoB-trnC* |
| 14 | 11 | 2 | 53,984 | 54,005 | TTTGTATAAGT | *ndhJ-ndhK* |
| 15 | 11 | 2 | 62,072 | 62,094 | TTTCAATTCTA | *rbcL-accD* |
| 16 | 11 | 2 | 76,843 | 76,872 | TATATTATATA | *clpP* intron |
| 17 | 11 | 2 | 94,992 | 95,013 | AAAAAAAAATG | *ndhF-rpl32* |
| 18 | 11 | 2 | 101,480 | 101,501 | TTCGAAACTAT | *ndhG-ndhI* |
| 19 | 11 | 2 | 104,037 | 104,058 | TTTCCTATAAA | *ndhA* intron |
| 20 | 11 | 2 | 122,394 | 122,415 | CTAATACTAAT | *trnV-rps12*^A^ |
| 21 | 12 | 2 | 7,240 | 7,260 | AGAAATATATAAATATAA | *trnK-trnQ* |
| 22 | 12 | 3 | 40,403 | 40,438 | TATTTTATAGAT | *psbZ-trnG* |
| 23 | 12 | 2 | 63,022 | 63,047 | AAACTCAAGAGA | *accD* gene |
| 24 | 12 | 5 | 63,758 | 63,815 | GGAATCTTATGA | *accD* gene |
| 25 | 12 | 2 | 83,301 | 83,324 | ATAAGAACATAA | *petD* intron |
| 26 | 12 | 2 | 90,444 | 90,471 | AATATATATATT | *rps3-rps19* |
| 27 | 12 | 2 | 135,602 | 135,625 | ATCTCTCTCAAT | *ycf2* gene^B^ |
| 28 | 13 | 2 | 8,815 | 8,841 | AAATTCTAAATAT | *psbI-trnS* |
| 29 | 13 | 2 | 11,641 | 11,667 | AATTAATAATATA | *trnR-atpA* |
| 30 | 14 | 2 | 55,984 | 56,012 | TAATATTTAGTTAT | *ndhC-trnV* |
| 31 | 14 | 2 | 78,560 | 78,588 | TTTTTATAGTGCAA | *clpP-psbB* |
| 32 | 15 | 2 | 11,591 | 11,621 | ATTTTTAATTAATTA | *trnR-atpA* |
| 33 | 15 | 2 | 64,698 | 64,729 | ATTGAATTTATATTA | *accD-psaI* |
| 34 | 15 | 2 | 74,043 | 74,077 | TATATAACATATAAT | *rpl33-rps18* |
| 35 | 15 | 2 | 135,917 | 135,946 | ACTAATTAAACCAAA | *trnI-trnH* |
| 36 | 18 | 2 | 128,436 | 128,471 | TATTTAGTTATTCAGTTA | *trnL-ycf2* |
| 37 | 21 | 2 | 76,109 | 76,150 | GATCATGAATTACTTATATTC | *rps12-clpP* |
| 38 | 21 | 3 | 76,764 | 76,847 | ATCAAATGTAAATGCTTATAT | *clpP* intron |
| 39 | 22 | 2 | 113,433 | 113,476 | CATATCTTTTTTGCTTTCTTTT | *trnN-trnR* |
| 40 | 24 | 3 | 63,675 | 63,745 | GAACCTTCTGAGAAATCTTATGAA | *accD* gene |
| 41 | 24 | 2 | 111,343 | 111,391 | TTTCTGTATCCAGACTAATACCAA | *ycf1* gene |
| 42 | 32 | 2 | 72,739 | 72,802 | TTGTATAACATATATTATAATAATAATAAAAG | *trnP-psaJ* |
| 43 | 36 | 2 | 63,415 | 63,506 | CTTATGAGGAACCTTCTGAGGAACCTTATTGGGAA | *accD* gene |
| 44 | 41 | 5 | 112,377 | 112,612 | TTTTTTTTCTTTTTGATTGTTATTTCTATATATGAAATTTCT | *ycf1-trnN* |
| 45 | 48 | 2 | 112,190 | 112,296 | ATTTCTATATTATTATATGAAATTTCTTTTTTTTTCTTTTTGATTGTT | *ycf1-trnN* |
| 46 | 60 | 3 | 63,487 | 63,697 | CTTATGAGGAACCTTCTGAGAAATCTTATGAGGAACCTTATTGGGAAC | *accD* gene |
| 47 | 60 | 2 | 69,118 | 69,237 | TTAATAATAATATAATTAATATATTCCTATTACAATTTATTACAATACAATATATTTAAT | *petA-psbJ* |
| 48 | 72 | 2 | 63,298 | 63,453 | ATTGGGAACCTTCTGAGAAATCTTATGAGGAACCTTCTGAGAAATCTTATGAGGAACCTTCTGAGGAACCTT | *accD* gene |

^A^ Repeat is also found in *Lotus japonicus, Millettia pinnata* and *Lupinus luteus*

^B^ Repeat is also found in *Millettia pinnata* and *Lupinus luteus*
