# Supplementary material for: Correction: The Complete Sequence of the Acacia ligulata Chloroplast Genome Reveals a Highly Divergent clpP1 Gene
Source: PLoS One. 2015 Sep 14;10(9):e0138367. doi: 10.1371/journal.pone.0138367 (PMC4569417; doi:10.1371/journal.pone.0138367)
Supplement: S3 Table — (DOCX) [file pone.0138367.s003.docx]

**S3 Table. Primers Used to Fill Gaps in the *Acacia ligulata* Chloroplast Genome Sequence.**

| **Name** | **Direction** | **Primer sequence (5’ to 3’)** | **Annealing temperature** |
| --- | --- | --- | --- |
| Gap_01 | Forward | GCGTATTTGCGTCTTTGATA | 64ºC |
|  | Reverse | ACAGATCGTATGGTAGGACA |  |
| Gap_02 | Forward | CGATTTCCTTCCCTATCAG | 49ºC |
|  | Reverse | CGATTTCCTTCCCTATCAG |  |
| Gap_03 | Forward | TCCGTTCCATGCCTCATT | 51°C |
|  | Reverse | CCACAACGACCGAATTAA |  |
| Gap_04 | Forward | TTGGGCGTTTATTACTTGGA | 50°C |
|  | Reverse | CTCATTATCAGTTGACAAGGTC |  |
| Gap_05 | Forward | TGGTGTTTCTAACCATCCAC | 51ºC |
|  | Reverse | GGAATTCGGATTGATGAACT |  |
| Gap_06 | Forward | TTTCTCAGATAACACTCAGA | 65ºC |
|  | Reverse | AAGAGAGGGGAGAGATCTTC |  |
| Gap_07 | Forward | ATCATGTCTTTCAAGTCGCA | 51ºC |
|  | Reverse | GGTTTTGGTCCCGCTATT |  |
| Gap_08 | Forward | TTTGTCAATCCCAGTCCAAA | 50ºC |
|  | Reverse | TTCAGGTAATTTCGCGAAGA |  |
| Gap_09 | Forward | CGGATTCCTATCTAACGATCC | 53ºC |
|  | Reverse | GGAATTAAGAAAAGAGGACCC |  |
| Gap_10 | Forward | GAGGTCTTCTAAACCTTTGG | 51ºC |
|  | Reverse | TCTTGTTGAGTTACGTGCTT |  |
| Gap_11 | Forward | TATTAAACCCGAAACTCCCG | 66ºC |
|  | Reverse | GCATACTAACTCGCCTTCTT |  |
| Gap_12 | Forward | TTTTCACGAGCCCATATG | 51ºC |
|  | Reverse | AAAGATTACCGGGGAATTGT |  |
| Gap_13 | Forward | AAAGTATATGAGCACTCCGG | 51ºC |
|  | Reverse | CACCTTTGGAAGTATTAAGGG |  |
| Gap_14 | Forward | GGGGTCAAACTTCTGGAAA | 51ºC |
|  | Reverse | GCGTCTTCTCTTTGGCAAA |  |
| Gap_15 | Forward | AGATCTACTCCTATGAATGTGG | 65ºC |
|  | Reverse | CTACGTCAGGATAACTCTTC |  |
| Gap_16 | Forward | TCCAGTAATTACCGTTCGTT | 65ºC |
|  | Reverse | GGATTAATTGTGCATCCAAC |  |
| Gap_17 | Forward | GGGCAAAAGAGTAATTGAGC | 65ºC |
|  | Reverse | GGTACCTCGATTTAATATTTGT |  |
| Gap_18 | Forward | GTAGAGTAGTCGACAAACAA | 65ºC |
|  | Reverse | TCAAAACATCTCTTCCTCGA |  |
| Gap_19 | Forward | GGGGATTTTGTGACATTTCG | 50ºC |
|  | Reverse | TCGTACGAGATAGAGGAACC |  |
| Gap_20 | Forward | ACGGCTCTACTATGGAATTG | 51ºC |
|  | Reverse | TGCCTTCGCCATATCAATAT |  |
